# Health, safety, and socioeconomic impacts of cannabis liberalization laws: An evidence and gap map

**DOI:** 10.1002/cl2.1362

**Published:** 2023-10-30

**Authors:** Eric L. Sevigny, Jared Greathouse, Danye N. Medhin

**Affiliations:** ^1^ Department of Criminal Justice and Criminology Georgia State University Atlanta Georgia USA

## Abstract

**Background:**

Globally, cannabis laws and regulations are rapidly changing. Countries are increasingly permitting access to cannabis under various decriminalization, medicalization, and legalization laws. With strong economic, public health, and social justice incentives driving these domestic cannabis policy reforms, liberalization trends are bound to continue. However, despite a large and growing body of interdisciplinary research addressing the policy‐relevant health, safety, and socioeconomic consequences of cannabis liberalization, there is a lack of robust primary and systematic research that comprehensively investigates the consequences of these reforms.

**Objectives:**

This evidence and gap map (EGM) summarizes the empirical evidence on cannabis liberalization policies. Primary objectives were to develop a conceptual framework linking cannabis liberalization policies to relevant outcomes, descriptively summarize the empirical evidence, and identify areas of evidence concentration and gaps.

**Search Methods:**

We comprehensively searched for eligible English‐language empirical studies published across 23 academic databases and 11 gray literature sources through August 2020. Additions to the pool of potentially eligible studies from supplemental sources were made through November 2020.

**Selection Criteria:**

The conceptual framework for this EGM draws upon a legal epidemiological perspective highlighting the causal effects of law and policy on population‐level outcomes. Eligible interventions include policies that create or expand access to a legal or decriminalized supply of cannabis: comprehensive medical cannabis laws (MCLs), limited medical cannabidiol laws (CBDLs), recreational cannabis laws (RCLs), industrial hemp laws (IHLs), and decriminalization of cultivations laws (DCLs). Eligible outcomes include intermediate responses (i.e., attitudes/behaviors and markets/environments) and longer‐term consequences (health, safety, and socioeconomic outcomes) of these laws.

**Data Collection and Analysis:**

Both dual screening and dual data extraction were performed with third person deconfliction. Primary studies were appraised using the Maryland Scientific Methods Scale and systematic reviews were assessed using AMSTAR 2.

**Main Results:**

The EGM includes 447 studies, comprising 438 primary studies and nine systematic reviews. Most research derives from the United States, with little research from other countries. By far, most cannabis liberalization research focuses on the effects of MCLs and RCLs. Studies targeting other laws—including CBDLs, IHLs, and DCLs—are relatively rare. Of the 113 distinct outcomes we documented, cannabis use was the single most frequently investigated. More than half these outcomes were addressed by three or fewer studies, highlighting substantial evidence gaps in the literature. The systematic evidence base is relatively small, comprising just seven completed reviews on cannabis use (3), opioid‐related harms (3), and alcohol‐related outcomes (1). Moreover, we have limited confidence in the reviews, as five were appraised as minimal quality and two as low quality.

**Authors’ Conclusions:**

More primary and systematic research is needed to better understand the effects of cannabis liberalization laws on longer‐term—and arguably more salient—health, safety, and socioeconomic outcomes. Since most research concerns MCLs and RCLs, there is a critical need for research on the societal impacts of industrial hemp production, medical CBD products, and decriminalized cannabis cultivation. Future research should also prioritize understanding the heterogeneous effects of these laws given differences in specific provisions and implementation across jurisdictions.

## 1 PLAIN LANGUAGE SUMMARY

### 1.1 The evidence on cannabis liberalization laws is unevenly distributed across policies and outcomes, and the research remains under‐reviewed

An evidence and gap map (EGM) provides a systematic and visual representation of available research on a specific issue or topic. This EGM presents evidence on the effects of cannabis policies. The map reveals areas of both evidence concentration, which can aid systematic review and policy decisions, and evidence gaps, which can inform research and funding priorities.

Studies examining the effects of medical and recreational cannabis laws on cannabis use are relatively common. The evidence base for other laws and outcomes remains underdeveloped.

### 1.2 What is this EGM about?

The map provides descriptive and interactive displays of empirical evidence on the effects of cannabis liberalization laws. Specific interventions include laws that create or expand access to cannabis by removing criminal penalties, allowing medical use, or legalizing use for adults. Main outcomes include health, safety, and socioeconomic factors that are potentially linked to cannabis liberalization.

### 1.3 What studies are included?

The EGM includes 447 studies, comprising 438 primary studies and nine systematic reviews. Eligible studies included works published in English since 1970 that employed quasi‐experimental designs.

### 1.4 What is the aim of this evidence and gap map (EGM)?

The aim of this EGM is to present evidence on the effects of cannabis liberalization laws, including laws that create or expand access to cannabis by removing criminal penalties, allowing medical use, or legalizing use for adults.

### 1.5 What are the main findings of this map?

Most cannabis liberalization research focuses on the effects of medical cannabis laws and recreational cannabis laws. These studies primarily investigate overall effects, but a sizable number also explore the specific effects of cannabis dispensaries.

Studies targeting other relevant laws—including medical cannabidiol laws, industrial hemp laws, and cultivation decriminalization laws—are relatively rare.

The map documented 113 distinct outcomes, with cannabis use outcomes investigated most frequently.

Although no geographic restrictions were applied, most of the evidence is from North America, predominantly the USA.

The systematic evidence base is relatively small, comprising just seven completed reviews on cannabis use, opioid‐related harms, and alcohol‐related outcomes. Five reviews were assessed as minimal quality and two as low quality.

### 1.6 How up to date is this EGM?

The literature search is current through 15 August 2020, with final study additions from other sources occurring on 12 November 2020.

## 2 BACKGROUND

### 2.1 The problem, condition, or issue

Globally, cannabis laws and regulations are rapidly changing. Since the late 1980s, countries have increasingly abandoned strict prohibition in favor of alternative approaches legalizing the use and sales of cannabis (Decorte, 2020). In the United States, as of 2022, 38 states have enacted medical cannabis laws authorizing use for qualifying health conditions, and 20 states have passed recreational cannabis laws legalizing home cultivation and/or retail sales for adult use (Klieger, 2017; Klitzner, 2017; Schauer, 2021). Dozens of other countries have also permitted access to cannabis under various decriminalization, medicalization, and legalization regimes (Belackova, 2020; Decorte, 2017; Rehm, 2019). Most notably, Uruguay became the first country to fully legalize recreational cannabis in 2013 (Queirolo, 2020), followed by Canada in 2018 (Fischer, 2018) and Malta in 2021 (Bubola, 2021).

With strong economic, public health, and social justice incentives driving domestic cannabis policy reforms (Newman, 2021; Oldfield, 2021), these trends will only accelerate. However, despite a large and growing body of interdisciplinary research addressing the policy‐relevant health, safety, and socioeconomic outcomes of cannabis liberalization, there remains a paucity of systematic research examining the potential benefits and harms of these reforms. In fact, outside of a handful of narrowly targeted narrative syntheses and meta‐analyses, no systematic reviews have fully scoped this diverse empirical literature. Consequently, this hampers policymakers’ ability to understand the impacts of these policies when adoptions or amendments are considered. This evidence and gap map (EGM) summarizes the empirical evidence on cannabis liberalization policies, highlights current knowledge gaps, and identifies opportunities for future research.

### 2.2 Why was it important to develop this EGM?

This EGM provides the first comprehensive review of the available evidence on cannabis liberalization reforms, capturing important heterogeneity in target populations, research designs, and study quality that is crucial to understanding this literature (Choo, 2017; Hunt, 2015; Pacula, 2017). Accordingly, the EGM offers stakeholders both a timely summary of the evidence and a roadmap for prioritizing future research.

### 2.3 Existing EGMs and/or relevant systematic reviews

Although there are no existing EGMs in this policy space, more than a dozen systematic reviews have been published examining the effects of cannabis liberalization policies on a small subset of relevant outcomes, namely cannabis use, other substance use, cannabis use disorders, roadway safety, and opioid‐related mortality.

Most systematic reviews assessed youth and adult cannabis use as the primary outcome, including one rapid review (O'Grady, 2022), four narrative reviews (Lachance, 2022; Leung, 2018; Pacula, 2022; Smart, 2019), and two meta‐analyses (Melchior, 2019; Sarvet, 2018). Harrell et al. (Harrell, 2022) also examined cannabis use as part of a larger scoping review of factors linked to vaping, and Pacula and colleagues (Pacula, 2022; Smart, 2019) investigated alcohol use both alone and in combination with cannabis, updating an earlier review by Guttmannova et al. (Guttmannova, 2016). While the reviews’ findings are mixed, two broad generalizations hold: increases in cannabis use, when observed, were more likely (i) among young adults than adolescents and (ii) following passage of recreational versus medical cannabis laws.

A smaller number of systematic reviews have investigated specific harms linked to cannabis liberalization. Several reviews examined cannabis use disorders, but none could draw firm conclusions (Leung, 2018; O'Grady, 2022; Smart, 2019). Similarly, a rapid review (Ansari, 2020), narrative review (Vyas, 2018), and meta‐analysis (Chihuri, 2019) found the overall evidence on opioid‐related mortality and associated harms to be inconsistent and inconclusive. A narrative review of roadway safety by Hasan et al. (Hasan, 2022) determined the evidence was insufficient to draw strong conclusions with respect to driving under the influence of drugs. However, a narrative review by Vingilis et al. (Vingilis, 2021) concluded, albeit cautiously, that cannabis commercialization was linked to increases in both traffic accidents and driver cannabis‐positivity rates.

In summary, existing systematic reviews of the effects of cannabis liberalization policies primarily focus on cannabis use, despite primary studies investigating a much broader and arguably more salient set of outcomes (Sznitman, 2015). Recent conceptual mappings across varied national contexts have identified dozens of performance indicators in the public health, community safety, and economic sectors that are relevant for understanding the full scope of cannabis policy impacts (Campeny, 2020; Fischer, 2018; Maslov, 2016).

### 2.4 EGMs: Definition and purpose

EGMs involve systematic searches of policies or interventions to identify gaps in knowledge and future research needs (Miake‐Lye, 2016). Results are typically presented in the form of a detailed graph and/or interactive database. Development of the current EGM adhered to published guidance (O'Leary, 2017; White, 2020).

## 3 OBJECTIVES

This EGM had three key objectives:

1. Develop a clear conceptual framework linking cannabis liberalization laws to associated health, safety, and socioeconomic outcomes.
2. Provide a structured summary of the empirical evidence, research setting and context, and study quality from existing primary empirical studies and systematic reviews for use by policymakers, researchers, and other stakeholders.
3. Identify study clusters that can support further systematic review, and document evidence gaps where additional primary research and funding resources are needed.

### 3.1 EGM framework

#### 3.1.1 Scope of the EGM

This EGM focuses on the impacts of laws and policies that expand legal access to cannabis. This encompasses medical, recreational, and industrial cannabis laws, as well as cannabis cultivation decriminalization laws that, while not legalizing production, abolish criminal penalties for small‐scale cultivation. The vertical axis of the map captures this policy dimension. The horizontal axis of the map captures health, safety, and socioeconomic outcomes that are theoretically or practically responsive to cannabis liberalization.

#### 3.1.2 Conceptual framework

**Figure 1:**
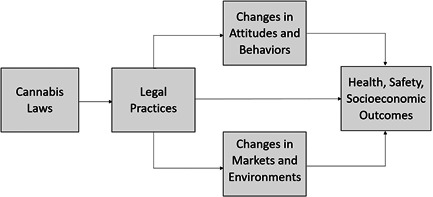
Conceptual framework of the effects of cannabis liberalization laws on health, safety, and socioeconomic outcomes.

Legal epidemiology is an interdisciplinary approach to studying causal effects of laws and policies on population‐level outcomes (Burris, 2013; Burris, 2016). Drawing upon this approach, Figure 1 frames our understanding and interpretation of cause‐and‐effect factors. The framework distinguishes policy from practice. Cannabis laws are statutes and regulations governing the production, availability, and use of cannabis. Legal practices reflect on‐the‐ground implementation of these laws. This distinction is important because the “law on the books” may deviate materially from the “law on the streets.” For example, it has typically taken one to 2 years post‐legalization for recreational cannabis markets to be fully open and operational in US states (Schauer, 2021).

The conceptual framework also highlights how laws and practices affect intermediate changes in attitudes and behaviors (e.g., perceptions of harm, frequency of use) and markets and environments (cannabis potency, availability of cannabis). These intermediate factors are themselves worthy of study, especially since ultimate outcomes (e.g., respiratory disease) take longer to manifest than more proximal factors (e.g., vaping prevalence).

#### 3.1.3 Stakeholder engagement

We solicited feedback on EGM concept and design from selected researchers and stakeholders in the areas of drug and cannabis policy from various organizations, including the International Society for the Study of Drug Policy, Global Cannabis Cultivation Research Consortium, and RAND Drug Policy Research Center. We received comments from multiple domain experts that helped improve the research protocol, including conceptualization and scope of the project, coding guide development, and selection of academic and gray literature database sources.

## 4 METHODS

### 4.1 Criteria for including and excluding studies

#### 4.1.1 Types of interventions

Eligible studies included policies that create or expand access to a legal or decriminalized supply of cannabis. As summarized in Table 1, this scope encompasses five distinct types of laws: comprehensive medical cannabis laws (MCLs) (e.g., Rehm, 2019), limited medical cannabidiol laws (CBDLs) (e.g., Alharbi, 2020), recreational cannabis laws (RCLs) (e.g., Klitzner, 2017), industrial hemp laws (IHLs) (e.g., McGregor, 2020), and decriminalization of cultivation laws (DCLs) (e.g., Belackova, 2020).

**Table 1:** Cannabis liberalization laws.

Law or Policy	Description
Medical cannabis law (MCL)	Grants qualified patients with a physician's recommendation or prescription the legal right to use and possess cannabis and, in some jurisdictions, cultivate cannabis to treat approved medical conditions.
Cannabidiol law (CBDL)	Grants qualified patients with a physician's recommendation or prescription the legal right to use and possess high‐CBD/low‐THC cannabis products, such as oils and tinctures, to treat approved medical conditions. Tetrahydrocannabinol (THC) is the main psychoactive constituent of cannabis, whereas cannabidiol (CBD) is a key nonpsychoactive compound.
Recreational cannabis law (RCL)	Legalizes adult cannabis use and regulates the modes of cannabis production and supply.
Industrial hemp law (IHL)	Legalizes the cultivation of low‐THC cannabis (e.g., <0.3% THC), otherwise known as hemp, to produce foods, supplements, and nonconsumable products such as textiles and cosmetics
Decriminalization of cultivation law (DCL)	Removes criminal penalties for small‐scale (e.g., <6 plants) home cultivation of cannabis for personal use and social supply

Studies of policies that do not explicitly increase the supply of cannabis meant for human consumption were considered out‐of‐scope. This delimitation excludes, for instance, studies of decriminalization laws that only remove penalties for cannabis use or possession, industrial hemp laws that legalize cannabis cultivation solely for producing textiles and other nonconsumable products, and other policies that do not establish clear regulatory authority over cannabis supply. Note that the latter exclusion applies to the US Department of Justice's Ogden Memorandum, which articulated a federal policy of nonenforcement in states with strong and effective medical cannabis regulations, but not to the Dutch “coffeeshop” model which de facto legalized the retail supply of cannabis under uniform and enforceable national regulations (Korf, 2020).

#### 4.1.2 Types of outcomes

Studies were included in the EGM if they measured health, safety, or socioeconomic outcomes. Health outcomes assess physical or mental well‐being and quality of life, safety outcomes measure levels of security and risk of harm, and socioeconomic outcomes capture social and economic conditions. We also included studies focusing on intermediate outcomes reflecting changes in structural conditions and incentives, including attitudes and behaviors (e.g., perceptions of marijuana, prevalence of use, routes of administration) and markets and environments (e.g., cannabis prices and potency, advertising). As our purview was purposely broad, we placed no a priori restrictions on specific outcomes within this framework, nor did we limit the measurement modality (e.g., self‐report, administrative data). Table 2 describes and provides examples of these eligible intermediate and ultimate outcomes.

**Table 2:** Intermediate and ultimate outcomes.

Outcomes	Description	Examples
*Intermediate*		
Attitudes and behaviors	Perspectives and activities of individuals	Perceptions of harm, prevalence of cannabis use, prevalence of other drug use, routes of administration
Markets and environments	Marketplace and contextual characteristics	Availability of cannabis, cannabis prices, and potency, product marketing and advertising
*Ultimate*		
Health	Physical or mental well‐being and quality of life	Substance use disorders, overdose, suicide, mortality, mental and physical health
Safety	Levels of security and risk of harm	Crime, drugged driving, traffic accidents, workplace safety
Socioeconomic	Social and economic conditions	Housing values, educational attainment, productivity, environmental consequences

#### 4.1.3 Types of target populations

RCLs target the general adult population, whereas MCLs and CBDLs target people who have been diagnosed with specific medical conditions (e.g., epilepsy, HIV, chronic pain). However, because cannabis liberalization research is often concerned with unintended consequences among groups not directly targeted by these laws (e.g., accidental ingestion by youth), we placed no a priori restrictions on study target or reference populations.

#### 4.1.4 Types of settings

Eligible study settings included any national or subnational jurisdiction that decriminalized, medicalized, or legalized the supply of cannabis since 1970, a starting year that encompasses all modern cannabis policy reforms. Studies not published in English were excluded because the study team had no fluency in other languages.

#### 4.1.5 Types of evidence

We included both completed and ongoing primary studies and systematic reviews in the EGM. We targeted all types of quasi‐experimental and experimental research designs, although we did not anticipate locating true experiments since cannabis policies are not randomly assigned to states. Quasi‐experimental designs use observational data to estimate the average effect of an intervention, where the comparator consists of groups and/or time periods that are not exposed to the policy. Eligible quasi‐experimental designs included but were not limited to difference‐in‐differences, interrupted time series, regression discontinuity, synthetic control method, instrumental variables, propensity score matching, controlled before‐and‐after, cohort design, and regression‐based cross‐sectional analyses that control for confounding variables (Bärnighausen, 2017; Reeves, 2017; Rockers, 2015). We excluded studies if they used only simulated data or forecasting techniques, were primarily descriptive in nature, or employed only qualitative evidence. We included systematic reviews of all types if the reviews reported replicable methods and summaries of impact (MacEntee, 2019).

### 4.2 Search methods for identification of studies

We searched academic databases and gray literature sources and implemented supplemental search strategies to locate relevant citations. All retrieved references were downloaded into EndNote software for bibliographic management.

#### 4.2.1 Academic databases

Between August 13–15, 2020, we searched the following 23 indexed academic databases and systematic review registries (with vendor noted):

- Academic Search Complete (EBSCO)
- APA PsycINFO (EBSCO)
- Applied Social Sciences Index and Abstracts (ProQuest)
- Campbell Library (Campbell Collaboration)
- CINAHL Plus (EBSCO)
- Cochrane Library (Wiley)
- Criminal Justice Abstracts (EBSCO)
- Criminal Justice Database (ProQuest)
- Dissertations & Theses A&I (ProQuest)
- EconLit (EBSCO)
- Embase (Elsevier)
- ERIC (EBSCO)
- Health Management/Administration Database (ProQuest)
- Health Source: Nursing/Academic Edition (EBSCO)
- International Bibliography of the Social Sciences (ProQuest)
- Legal Source (EBSCO)
- Medline (EBSCO)
- National Criminal Justice Reference Service (ProQuest)
- PAIS Index (ProQuest)
- PROSPERO (University of York)
- Research Library (ProQuest)
- ScienceDirect (Elsevier)
- Web of Science: Core Collection (Clarivate Analytics)

We used both natural language and controlled vocabulary terms to search databases using Boolean logic across three domains: (1) cannabis, (2) policy change, (3) and quantitative methods. Natural language terms were used to query title, abstract, and keyword fields. Controlled vocabulary terms were used to query subject fields. If the database search engine allowed, we automatically expanded the search to include related words and equivalent subjects, while delimiting the query by language (English‐only), time (since 1970), and source (e.g., no commentaries). See Supporting Information: Appendix 1 for the complete database search strategy.

#### 4.2.2 Gray literature sources

Between August 13–15, 2020, we also searched the following 11 gray literature sources:

- Don M. Gottfredson Library of Criminal Justice Gray Literature Database (https://njlaw.rutgers.edu/cj/gray/index.php)
- European Monitoring Centre for Drugs and Drug Addiction (https://www.emcdda.europa.eu/publications)
- International Society for the Study of Drug Policy Grey Literature Bibliography (https://www.issdp.org/bibliography-introduction/)
- IZA – Institute of Labor Economics (https://www.iza.org/publications)
- National Bureau of Economic Research Working Papers (https://www.nber.org/papers.html)
- RAND Corporation (https://www.rand.org/search/advanced-search.html)
- National Drug & Alcohol Research Centre (https://ndarc.med.unsw.edu.au/resources)
- Open Society Framework (https://osf.io/preprints/)
- RePEc (https://ideas.repec.org/)
- ScholarWorks (https://scholarworks.gsu.edu/)
- Social Sciences Research Network (https://papers.ssrn.com/sol3/DisplayAbstractSearch.cfm)

The search strategy for gray literature was simplified to accommodate their less sophisticated search engines, which commonly disallowed the use of wildcards, delimiters, or more advanced search logic.

#### 4.2.3 Supplementary search strategies

We augmented our systematic search by including studies from our personal libraries. We also reached out to subject matter experts for new, updated, or unpublished research. Finally, to identify additional potentially relevant studies, we (i) reviewed the references of eligible studies and reviews and (ii) used Google Scholar to forward citation search for eligible studies. The final addition to our pool of potentially eligible studies from any of these supplemental sources was entered on November 12, 2020, although we continued to update the bibliographic information of working papers and prepublication articles as we were alerted to newer versions of record.

### 4.3 Study screening and selection

Before screening, duplicate records were removed using the EndNote deduplication protocol outlined by Bramer et al. (Bramer, 2016). We screened both titles/abstracts and full‐text using the DistillerSR platform (Evidence, 2022). All three authors took part in the study screening and selection process. Initially, the titles and abstracts of retrieved records were independently screened by two reviewers according to the following three criteria:

1. Does the study examine a cannabis liberalization law or policy that expands legal access to cannabis supply for personal consumption?
2. Does the study examine (i) an intermediate market/environmental or attitudinal/behavioral outcome or (ii) a final health, safety, or socioeconomic outcome?
3. Is the study (i) a systematic review or (ii) does it employ a quasi‐experimental study design providing quantitative evidence of impact?

Studies were passed to the full‐text screening stage if all three questions were answered affirmatively. Screening conflicts were resolved by reaching consensus with a third reviewer. To reduce screening workload at the title/abstract stage, we used DistillerSR's continuous reprioritization feature, which employs artificial intelligence to reorder unscreened studies based on their predicted relevance, coupled with the software's auditing and simulation tools to help identify potentially incorrectly excluded and incorrectly included studies (Hamel, 2020). Next, two reviewers independently and manually examined the full texts of all potentially eligible studies according to the same three screening questions (i.e., dual full‐text screening), with any conflicts arbitrated by a third reviewer. Any additional reasons why a study was deemed ineligible (e.g., superseded version, non‐English, full‐text unavailable) were also recorded at this stage.

### 4.4 Data collection and analysis

#### 4.4.1 Data extraction, coding, and management

Initial data extraction was performed independently by two coders using DistillerSR to collect study‐level descriptive data. Discrepancies were deconflicted by consensus, with a third reviewer arbitrating when needed. We coded bibliographic information, study setting and data years, publication type, funding information, policies and provisions, outcomes, study design, and study quality. When study‐level information was missing on any of these items, we coded it as “not reported.” If study criteria fell across multiple coding categories, we recorded all statuses. For instance, if a study used multiple research designs (e.g., difference‐in‐differences and instrumental variables) or study data spanned multiple decades (e.g., 1990–1999 and 2000–2009), each condition was coded in the final map. After this step, the original coding scheme was refined and adjusted to produce a final coding scheme for use with EPPI‐Reviewer (Thomas, 2022), the software platform we used to develop the online EGM. The final coding guide is presented in Supporting Information: Appendix 2.

#### 4.4.2 Quality appraisal

All studies were assessed for quality. For primary intervention studies, we used a modified version of the Maryland Scientific Methods Scale (SMS) (Madaleno, 2016; Sherman, 2002), a 5‐point scale ranging from 1 (*lowest quality*) to 5 (*highest quality*). SMS robustness is determined by the study's ability to identify a causal effect according to the following design criteria:

- Level 1: Study lacks either a comparison group or pre‐post observations on the outcome. These studies typically employ comparative cross‐sectional designs or one‐group pretest–posttest designs.
- Level 2: Study uses both a comparison group and pre‐post observations on the outcome. These studies may control for confounders or use propensity score matching, but do not account for unobservable differences between groups. Studies at this level include cohort designs, in which subjects allocate themselves to treatment status, and controlled before‐and‐after designs, in which subjects are nonrandomly allocated to treatment status.
- Level 3: Study employs (i) panel data to compare treated and comparison groups on many pre‐ and postintervention observations (e.g., difference‐in‐differences, synthetic control method, proportional hazard model) or (ii) time series data to compare a treated group across many timepoints both before and after an intervention (e.g., interrupted time series).
- Level 4: Study achieves quasi‐randomization by employing specific design features that mitigate selection bias and other forms of endogeneity (e.g., instrumental variables estimation, regression discontinuity).
- Level 5: Study randomizes subjects into treatment and control conditions (e.g., RCT).

If a study reported multiple designs (e.g., in a supplemental analysis), we coded the highest study quality level. Note that because no RCT studies have been conducted in this policy domain, we used only the first four quality levels. Accordingly, we adopted the following study quality descriptors for this EGM: Level 1 = minimal, Level 2 = low, Level 3 = moderate, and Level 4 = high. Being largely design‐driven and focused on internal validity, the SMS does not explicitly account for implementation quality nor does it assess other relevant dimensions of bias (e.g., confounding, selective effect size reporting). As such, the SMS has serious limitations as a comprehensive quality appraisal tool, but alternative risk of bias instruments are ill‐suited for the types of designs encountered in this review (Waddington, 2017).

For systematic reviews, we assessed risk of bias using AMSTAR 2 (Shea, 2017). We analogously summarized overall confidence in the results of each review according to AMSTAR 2 scoring criteria: Minimal (more than one critical flaw with or without noncritical weaknesses), low (one critical flaw with or without noncritical weaknesses), moderate (more than one noncritical weakness), and high (no more than one noncritical weakness).

### 4.5 Analysis and presentation

The EGM is presented as an interactive policy‐outcome matrix (https://eppi.ioe.ac.uk/cms/Portals/35/Maps/Cannabis_Liberalization_Laws_EGM), which is supplemented by a data visualization tool (https://eppi.ioe.ac.uk/eppi-vis/login/open?webdbid=243) as well as the descriptive analyses reported in this paper. The unit of analysis is a systematic review or primary study, represented as an entry within a cell of the EGM. If a review or study investigated multiple policies and/or outcomes, then it is reported in each relevant cell of the EGM.

We used Stata/MP 17.0 (StataCorp, 2021) or Microsoft Excel to produce the statistics and graphs. The online, interactive EGM was developed using EPPI‐Mapper (Digital, 2022). Cannabis liberalization policies form one axis of the map; health, safety, and socioeconomic outcomes form the other axis. Study entries within each cell of the interactive EGM are stratified and color‐coded by study quality (i.e., minimal to high), with additional selectable study filters including country, data period, publication type, funding information, policy and outcome sources, reference population, and research design.

## 5 RESULTS

### 5.1 Results of the search

**Figure 2:**
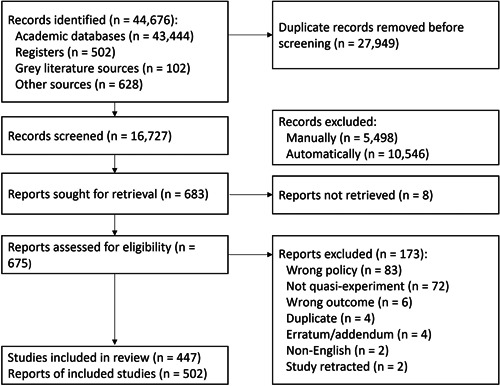
PRISMA flowchart.

Figure 2 depicts the PRISMA flow diagram for our literature search. The search strategy yielded 44,676 records, including 43,444 from academic databases, 502 from protocol registers, 102 from gray literature source, and 628 from other sources. After removing 27,949 duplicates, we retained 16,727 records for title and abstract review. Using DistillerSR's continuous reprioritization feature, we manually reviewed 5498 titles/abstracts (32.9%), stopping when the predicted likelihood of any remaining unscreened records being eligible for inclusion was less than 3%. At this point, we additionally reviewed the studies with the highest predicted likelihood of being either incorrectly included or incorrectly excluded, ultimately determining that no studies were wrongly classified. Overall, we manually excluded 5498 records and automatically excluded 10,546 by machine learning.

We sought 683 reports for retrieval, but could not locate the full texts for eight potentially eligible studies. Of the remaining 675 full‐text reports assessed for eligibility, we included 447 studies in the EGM, comprising 438 primary studies and nine systematic reviews. Overall, we retrieved 502 reports for the 447 eligible studies. The remaining 173 ineligible studies were excluded for the following reasons: wrong policy (83), wrong outcome (6), not quasi‐experimental (72), duplicate (4), erratum/addendum (4), non‐English (2), and study retracted (2).

### 5.2 Aggregate map

In this section, we discuss the major policy and outcome groupings in the EGM. More granular policy and outcome drill‐downs are presented in subsequent sections. Additional information on all eligible studies can be viewed and filtered in the online map (https://eppi.ioe.ac.uk/cms/Portals/35/Maps/Cannabis_Liberalization_Laws_EGM) and data visualizer (https://eppi.ioe.ac.uk/eppi-vis/login/open?webdbid=243).

Table 3 shows the aggregate number of primary studies (*n* = 438) and systematic reviews (*n* = 9) by major policy and outcome categories. On the policy axis, most studies in the EGM, whether primary studies or systematic reviews, focused on MCLs (*n* = 282) and RCLs (*n* = 248), with just a handful of studies investigating CBDLs (Aranda, 2020; Phillips, 2017; Prestemon, 2019; Shi, 2019; Shover, 2019), IHLs (Carrieri, 2019; Carrieri, 2020), or DCLs (Cerveny, 2017; Damrongplasit, 2010; Melchior, 2019; Williams, 2004). On the outcome axis, more than half the studies in the EGM investigated attitudes/behaviors (*n* = 232), about a quarter examined health (*n* = 123) and safety (*n* = 119), and roughly a 10th focused on markets/environments (*n* = 44) and socioeconomics (*n* = 48).

**Table 3:** Aggregate map: Number of studies by policy and outcome categories.

	Attitudes and behaviors	Markets and environments	Health	Safety	Socioeconomic	Row total
Medical cannabis law	142 | 9 | 151	22 | 1 | 23	81 | 6 | 87	73 | 1 | 74	29 | 0 | 29	273 | 9 | 282
CBD law	1 | 0 | 1	1 | 0 | 1	3 | 0 | 3	0 | 0 | 0	1 | 0 | 1	5 | 0 | 5
Recreational cannabis law	120 | 7 | 127	24 | 1 | 25	53 | 5 | 58	68 | 1 | 69	28 | 0 | 28	241 | 7 | 248
Industrial hemp law	1 | 0 | 1	1 | 0 | 1	0 | 0 | 0	1 | 0 | 1	0 | 0 | 0	2 | 0 | 2
Decriminalization of cultivation law	3 | 1 | 4	0 | 0 | 0	0 | 0 | 0	0 | 0 | 0	0 | 0 | 0	3 | 1 | 4
Column total	223 | 9 | 232	43 | 1 | 44	117 | 6 | 123	119 | 1 | 120	48 | 0 | 48	438 | 9 | 447

*Note*: The first figure in each cell refers to the number of primary studies, the second figure to the number of systematic reviews, and the third figure to the total number of studies.

Looking specifically at primary studies, the most populated cells in the map aligned in three tiers across both MCLs and RCLs. MCL research on attitudes/behaviors was predominant (*n* = 142), followed by studies addressing health (*n* = 81) and safety (*n* = 72) outcomes, and, lastly, socioeconomic (*n* = 29) and market/environment (*n* = 22) factors. Research on RCLs followed a similar overall pattern. Most RCL studies investigated attitudes/behaviors (*n* = 120), followed by research on matters of safety (*n* = 67) and health (*n* = 53), and trailed by studies on socioeconomics (*n* = 28) and markets/environments (*n* = 24). The number of primary studies across cells corresponding to CBDLs, IHLs, and DCLs was relatively sparse, reflecting the nascent policy environment and emergent nature of research on these other cannabis laws.

Comparatively, the seven completed and two ongoing systematic reviews in the EGM focused primarily on attitudes/behaviors and health responses to MCLs and RCLs. Few systematic reviews targeted other policies and outcomes. The exceptions are Melchior et al. (Melchior, 2019), who reported on the effects of DCLs on adolescent and young adult cannabis use, and a preregistered review by Busse et al. (Busse, 2018), who identified safety‐related motor vehicle accidents as a key outcome.

### 5.3 Description of included primary studies

#### 5.3.1 Study setting

Of the 438 primary studies in the EGM, most (*n* = 427) were conducted in North America, predominately the United States (*n* = 422) but also Canada (*n* = 10) and Mexico (*n* = 1). Five of the North American studies were conducted in the context of both the United States and Canada (Goodman, 2019; Goodman, 2020; Hildebrand, 2020; Lane, 2019; Rup, 2020), and one study examined spillover effects of MCLs in the United States on homicide in Mexico (Gavrilova, 2019). Studies from other countries were represented sparingly in the EGM: Australia (Damrongplasit, 2010; Williams, 2004), Czech Republic (Cerveny, 2017), Italy (Carrieri, 2019; Carrieri, 2020), Netherlands (Wouters, 2009; Wouters, 2012), and Uruguay (Jorge, 2020; Laqueur, 2020; Nazif‐Munoz, 2020).

**Figure 3:**
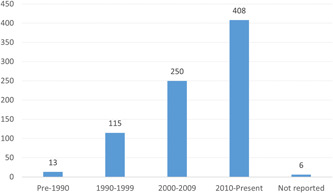
Number of primary studies by data coverage.

Figure 3 presents the number of primary studies by years of data coverage, aggregated by decade. Few studies (*n* = 13) presented or analyzed data from pre‐1990. However, the number of studies increases steadily across data years, with most (*n* = 408) reporting data analytic results, in part or whole, for the decade covering 2010‐Present. Six studies did not report the data period.

#### 5.3.2 Policies and provisions

As described previously, cannabis liberalization laws vary considerably in their overarching policy objectives. Most primary studies in the EGM (*n* = 358) focused on a single type of law. Among the remaining studies that examined multiple laws, the majority (*n* = 77) targeted MCLs and RCLs, with one examining MCLs and CBDLs and four investigating MCLs, RCLs, and CBDLs together.

Table 4 presents the number of primary studies by year of publication and policy. During the first eight publication years covered by the EGM (2004–2011) only eight studies were produced, with the very first study by Williams (Williams, 2004) examining the effect of Australian state decriminalization of cannabis consumption and cultivation on cannabis use. The earliest MCL study similarly examined effects on cannabis use in the United States (Gorman, 2007), whereas the initial RCL study compared cannabis buying behavior by “coffeeshop” availability across Netherlands cities (Wouters, 2009). The later publication of seminal studies on CBDLs (Phillips, 2017) and IHLs (Carrieri, 2019) reflects their more recent policy diffusion. As Table 4 shows, the pace of cannabis policy research accelerated in 2014, with the number of MCL studies predominating (77%) before 2019 but shifting primarily to RCLs (72%) since 2019.

**Table 4:** Number of primary studies by year of publication and policy.

Year	Medical cannabis law	Cannabidiol law	Recreational cannabis law	Industrial hemp law	Decrim of cultivation law	Total
2004	0	0	0	0	1	1
2005	0	0	0	0	0	0
2006	0	0	0	0	0	0
2007	1	0	0	0	0	1
2008	0	0	0	0	0	0
2009	0	0	1	0	0	1
2010	2	0	0	0	1	3
2011	2	0	0	0	0	2
2012	7	0	1	0	0	8
2013	4	0	0	0	0	4
2014	18	0	1	0	0	19
2015	21	0	8	0	0	25
2016	34	0	9	0	0	42
2017	34	1	24	0	1	47
2018	41	0	35	0	0	66
2019	58	3	78	1	0	114
2020	38	0	70	1	0	91
2021	12	1	13	0	0	21
2022	1	0	1	0	0	2

Policy design and implementation also differs substantially within major law types. Certain MCL jurisdictions, for instance, allow retail dispensaries and home cultivation, whereas others do not. Understanding how such policy heterogeneity affects outcomes is an important, and often overlooked, aspect of cannabis liberalization research (Pacula, 2014). Table 5 shows the number of primary studies by specific MCL and RCL policy provisions. Other cannabis liberalization policies are not presented in the table because no existing studies have investigated their specific policy dimensions.

**Table 5:** Number of primary studies by policy provisions.

Policy provisions	Medical cannabis law	Recreational cannabis law
Total studies	273	241
General law—only studies	144	140
Specific policy provision studies	129	101
Registries	34	0
Qualifying medical conditions	17	n/a
Employment protections	1	0
Caregiver regulations	1	n/a
Home cultivation	37	4
Plant/possession limits	4	1
Social clubs/collectives	4	0
Retail outlets	102	99
Product and marketing regulations	1	0
Policy provision index	10	1

Most MCL studies (*n* = 144) examined general law‐only effects, that is, whether the law was generally active and operational in a jurisdiction. Among the remaining MCL studies that explored the effects of one or more specific policy provisions, the majority focused on retail outlets (*n* = 102) followed by home cultivation (*n* = 37), registries (*n* = 34), and qualifying medical conditions (*n* = 17). Other MCL policy provisions (i.e., social clubs/collectives, plant/possession limits, product/marketing regulations, employment protections, and caregiver regulations) were infrequently investigated. Lastly, rather than focus on specific policy provisions, 10 studies operationalized policy indexes as measures of overall medical program leniency or restrictiveness.

As shown in Table 5, most RCL studies (*n* = 140) also examined general law‐only effects. Nearly every study that focused on an RCL policy provision identified the effects of retail outlets or dispensaries (*n* = 99). Other RCL policy provisions, including home cultivation and plant/possession limits, were rarely investigated.

Our analysis of cannabis policies and provisions reveals that the research tends to be unidimensional, largely focusing on MCLs and RCLs. Moreover, despite including a broad range of cannabis policy provisions in our coding guide, outside of a few key dimensions (e.g., retail outlets, home cultivation), many aspects of these laws have rarely, if ever, been studied.

#### 5.3.3 Policy data sources

**Figure 4:**
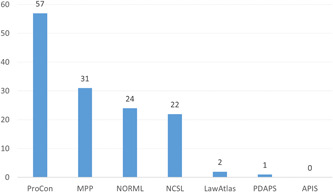
Number of primary studies by policy source/database.

Comparative policy research requires that statutes and laws be reliably and consistently coded across jurisdictions (Ritter, 2016). Although most study authors either developed their own policy indicators or did not report this information, Figure 4 depicts the number of studies for which specific policy data sources were reported, all of which describe policies across US states.

Of the 105 studies that drew upon one or more policy data source, most referenced ProCon (*n* = 57), an online encyclopedic resource for debatable policy issues. Special interest groups were also frequently sourced for policy data, including the Marijuana Policy Project (MPP; *n* = 31), National Organization for the Reform of Marijuana Laws (NORML; *n* = 24), and National Conference of State Legislatures (NCSL; *n* = 22). By comparison, academic and governmental sources were rarely referenced—including LawAtlas (*n* = 2), Prescription Drug Abuse Monitoring System (PDAPS; *n* = 1), and Alcohol Policy Information System (APIS; *n* = 0)—despite each source producing well‐documented cannabis policy data systems.

#### 5.3.4 Outcomes

For this EGM, we recorded a total of 113 unique outcomes across five domains: attitudes and behaviors (*n* = 19), markets and environments (*n* = 11), health (*n* = 37), safety (*n* = 24), and socioeconomic outcomes (*n* = 22). Multiple outcomes, both within and across domains, were commonly investigated within a single study.

**Figure 5:**
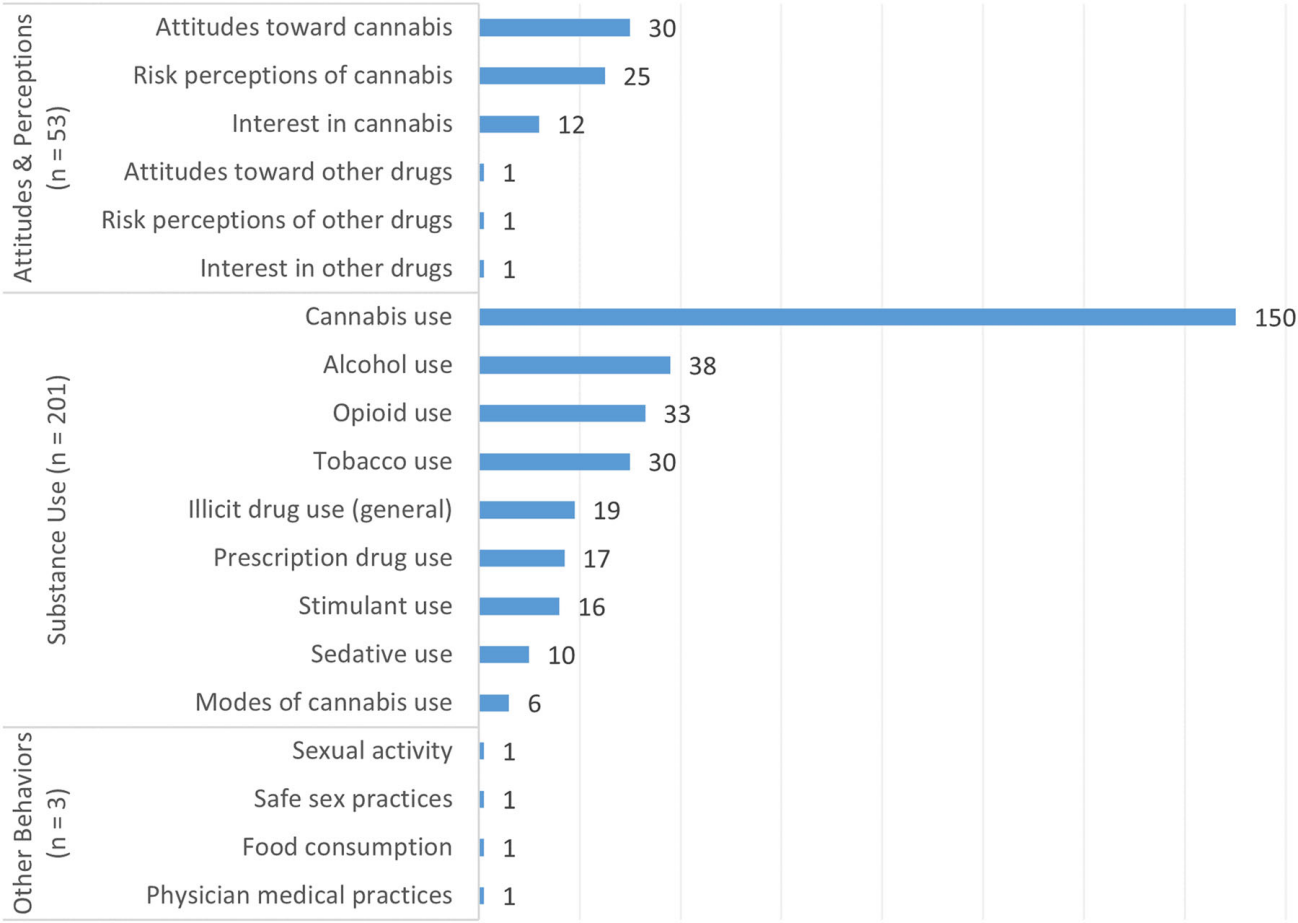
Number of primary studies (*n* = 223) by attitude and behavior outcomes.

Figure 5 presents the outcome distribution of 223 primary studies that investigated specific attitudes and behaviors. Most studies in this domain (*n* = 201) targeted substance use outcomes generally, with cannabis use (*n* = 150) the single most common outcome—investigated by about four times as many studies as alcohol use (*n* = 38). In the attitudes/perceptions subdomain (*n* = 53), cannabis‐related outcomes predominated, including attitudes toward cannabis (*n* = 30), risk perceptions of cannabis (*n* = 25), and interest in cannabis (*n* = 12). Lastly, a catch‐all subdomain of other behaviors (e.g., sex and junk food consumption) captured four outcomes across just three studies.

**Figure 6:**
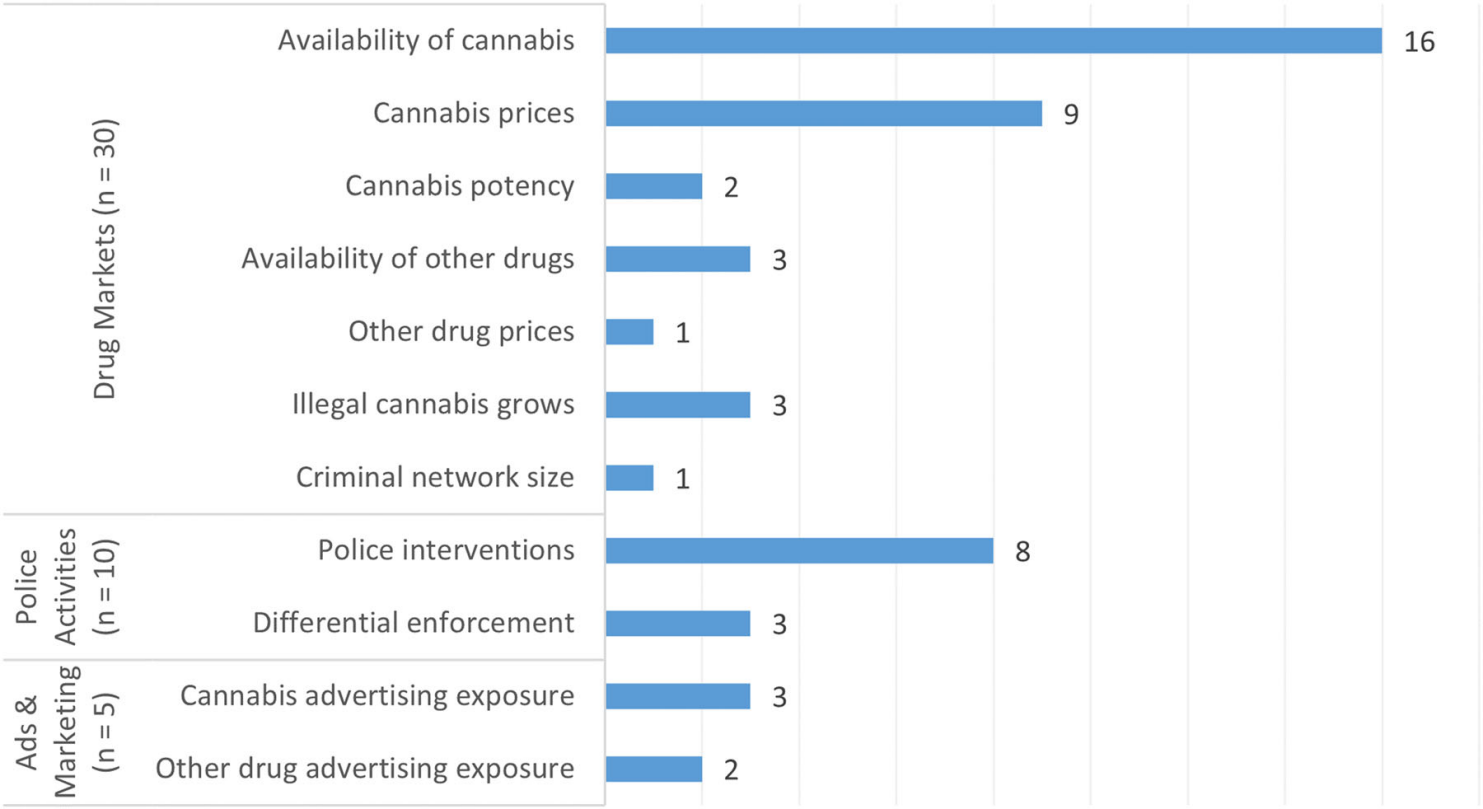
Number of primary studies (*n* = 43) by markets and environments outcomes.

The distribution of market and environment outcomes, reported across 43 primary studies, is presented in Figure 6. Features of drug markets were most common (*n* = 30), including studies on the availability of cannabis (*n* = 16) and cannabis prices (*n* = 9). Police activities were the focus of 10 studies, most of which addressed specific police interventions (*n* = 8) such as traffic stops and contraband seizures. Lastly, just five studies researched advertising and marketing outcomes, mainly involving cannabis advertising exposure (*n* = 3).

**Figure 7:**
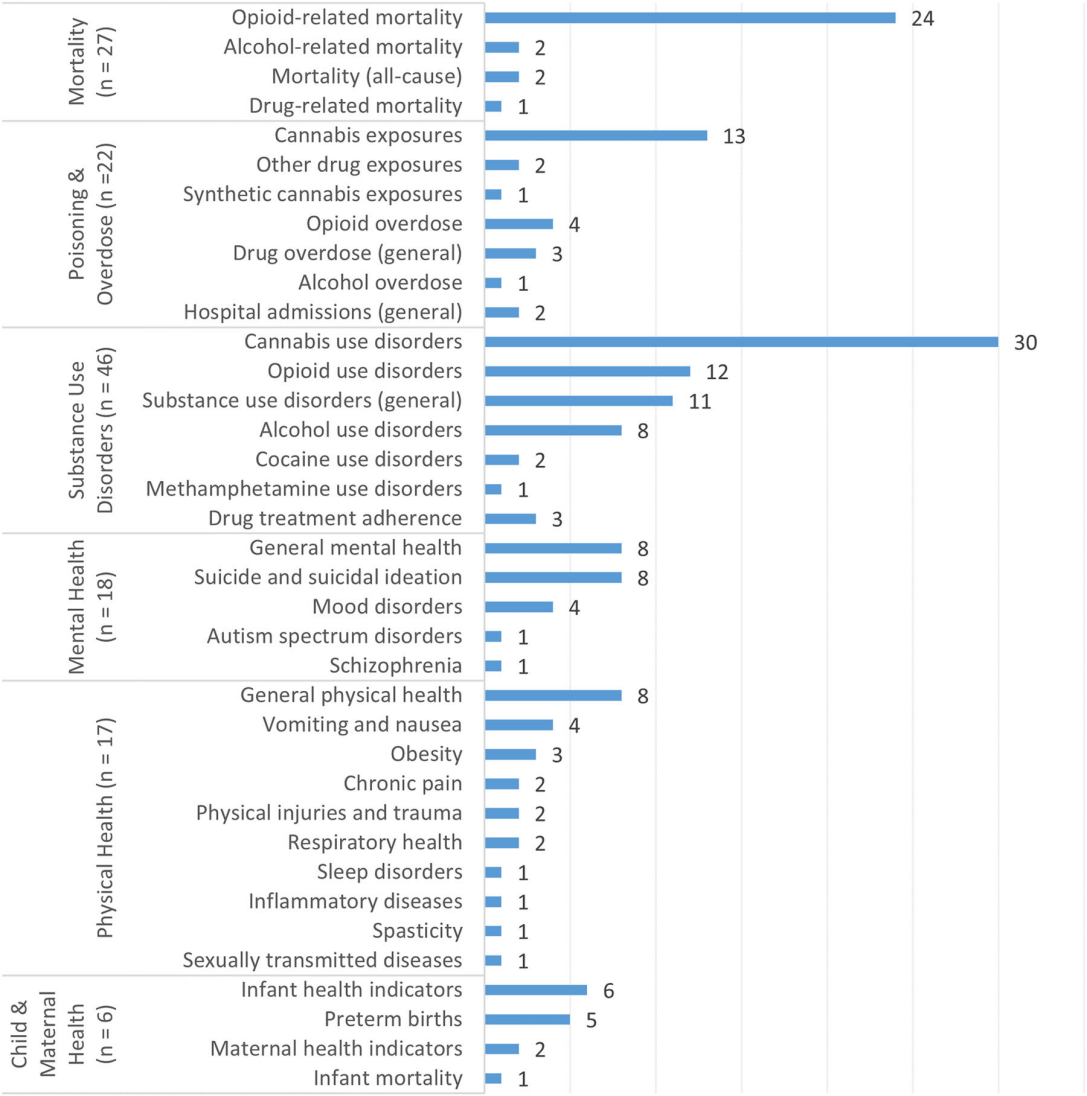
Number of primary studies (*n* = 117) by health outcomes.

As shown in Figure 7, cannabis policy researchers have investigated more than three dozen specific health outcomes across six subdomains and 117 primary studies: substance use disorders (*n* = 46), mortality (*n* = 27), poisoning and overdose (*n* = 22), physical health (*n* = 17), mental health (*n* = 18), and child and maternal health (*n* = 6). Not surprisingly, across all health outcomes, studies commonly targeted cannabis‐specific consequences, including cannabis use disorders (*n* = 30) and inadvertent cannabis exposures (*n* = 13). Given interest in the substitutability of cannabis for opioids in the treatment of certain health conditions, investigators also regularly studied opioid‐specific outcomes, including opioid‐related mortality (*n* = 24), opioid use disorders (*n* = 12), and opioid overdose (*n* = 4). However, most of the specific health outcomes we recorded (23 of 37, or 62%) were backed by just three or fewer studies.

**Figure 8:**
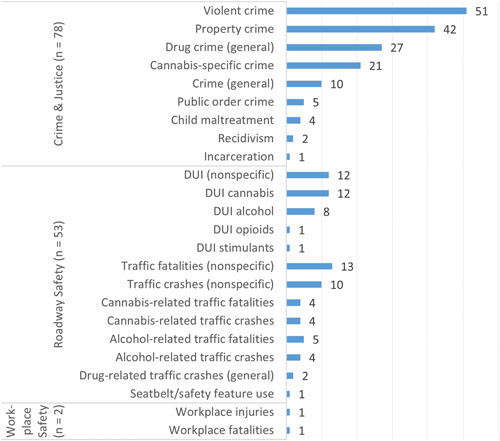
Number of primary studies (*n* = 119) by safety outcomes.

The number of studies investigating safety outcomes, presented in Figure 8, is divided into crime and justice (*n* = 78), roadway safety (*n* = 53), and workplace safety (*n* = 2) subdomains. Most studies on crime and justice issues focused on violent crime (*n* = 51), property crime (*n* = 42), and general drug crime (*n* = 27). Cannabis‐specific crimes, typically measured by cannabis arrests, were targeted less often by researchers. By comparison, other crime and criminal justice outcomes were infrequently investigated. Roadway safety studies mostly examined DUI cannabis (*n* = 12) and nondrug‐specific metrics of DUI (*n* = 12), traffic crashes (*n* = 10), and traffic fatalities (*n* = 13). Workplace safety issues, including injuries and fatalities occurring on the job, were rarely studied.

**Figure 9:**
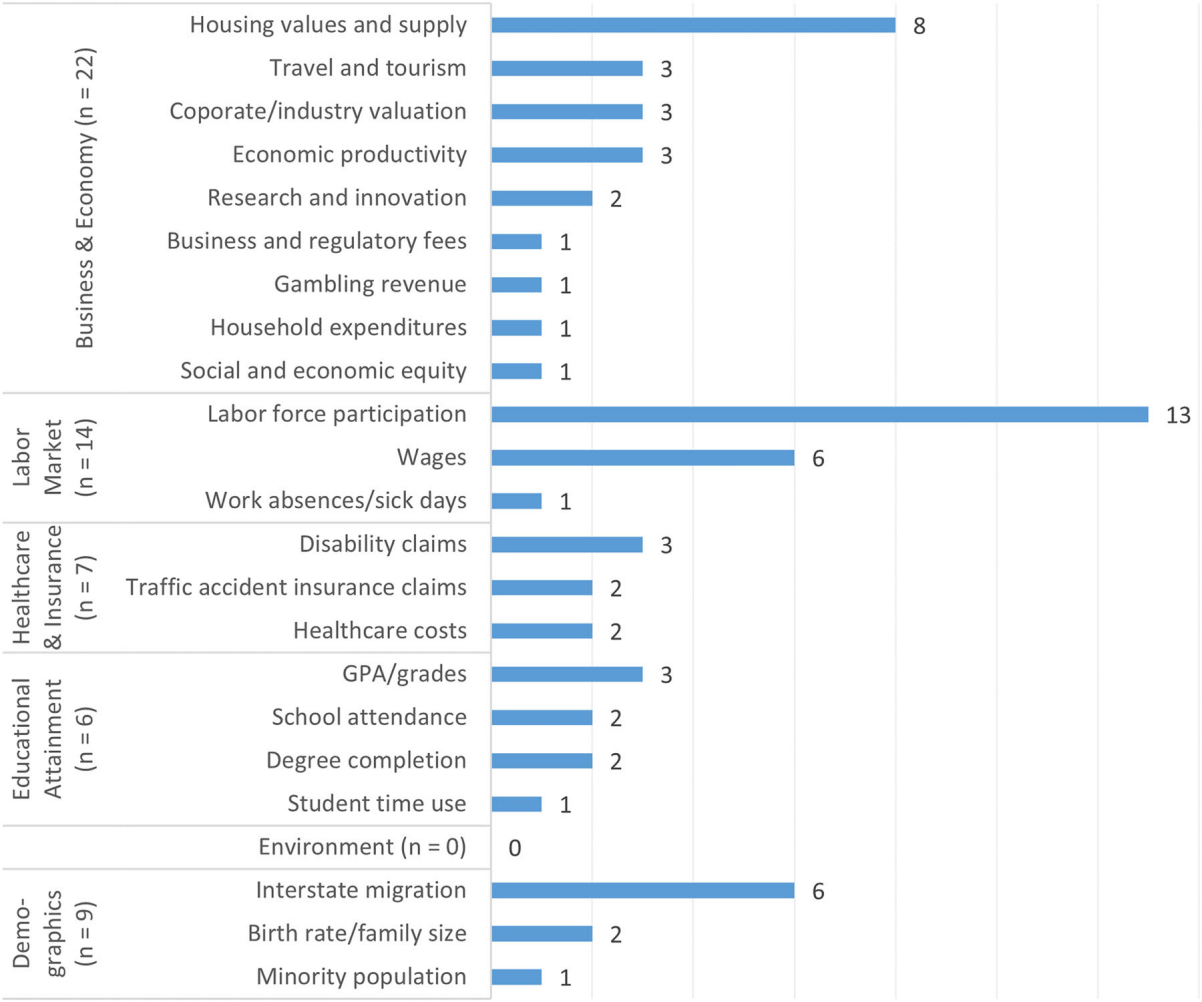
Number of primary studies (*n* = 48) by socioeconomic outcomes.

Figure 9 presents the outcome distribution of the 48 studies in the EGM that investigated socioeconomic outcomes, split across the following subdomains: business and economy (*n* = 22), labor market (*n* = 14), demographics (*n* = 9), healthcare and insurance (*n* = 7), and educational attainment (*n* = 6). Notably, no studies investigated environmental outcomes. The modal study in the business and economy sector addressed changes in housing values/supply (*n* = 8), whereas nearly every labor market study (*n* = 13) examined labor force participation (e.g., unemployment rate). Disability claims (*n* = 3), GPA/grades (*n* = 3), and interstate migration (*n* = 6) were the most common outcomes examined across the remaining three socioeconomic subdomains.

Overall, cannabis use was the most common outcome identified in this EGM, having been investigated in nearlyn two‐thirds (34%) of the included studies. More broadly, across five domains and 21 subdomains, we documented 113 distinct outcomes that have been investigated in the cannabis liberalization literature since 2004. Given that more than half (58%) the outcomes documented in this EGM were investigated by three or fewer studies, the evidence base remains overwhelmingly slim in many areas.

#### 5.3.5 Outcome data sources

**Figure 10:**
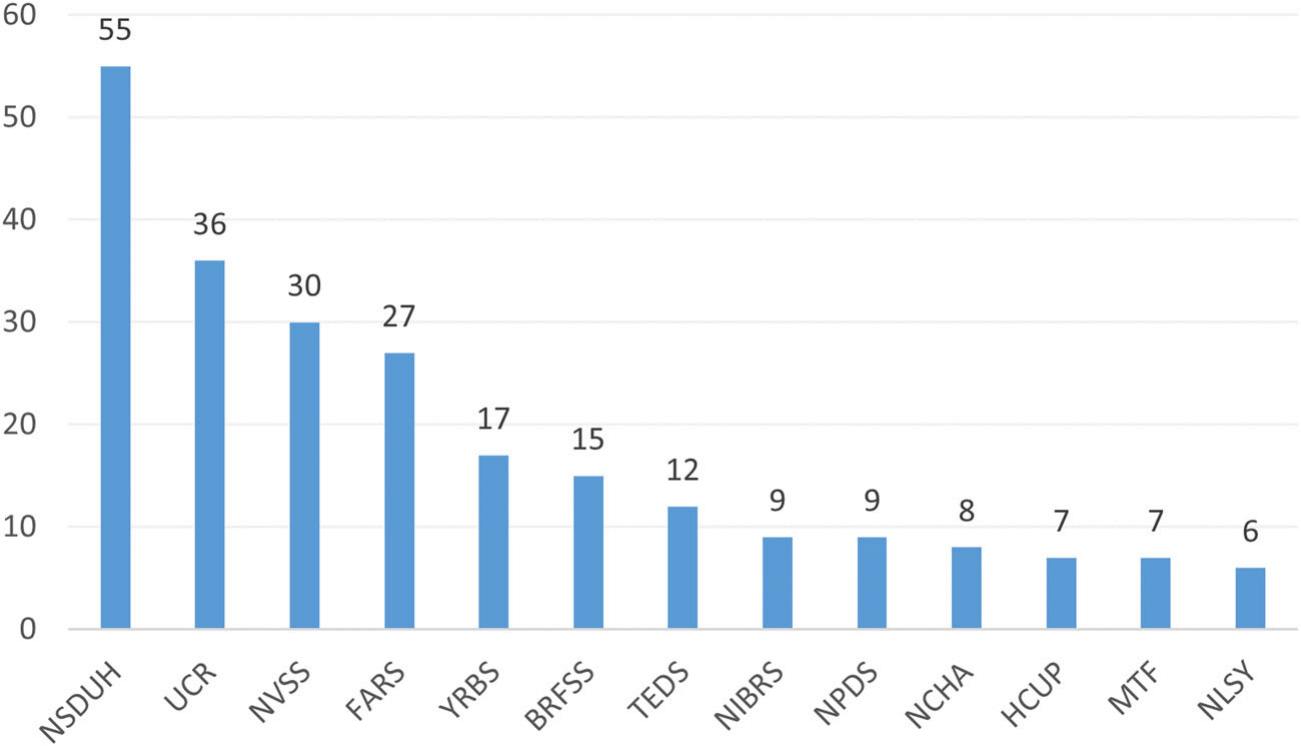
Number of primary studies by outcome data source.

While more than half the studies in this EGM collected primary data, used proprietary data sources, or analyzed other public data, many studies operationalized key outcomes from federally sponsored US serial data collections. As shown in Figure 10, the National Survey on Drug Use and Health (NSDUH) was employed most frequently (*n* = 55), followed by the Uniform Crime Reports (UCR; *n* = 36), National Vital Statistics System (NVSS; *n* = 30), Fatality Analysis Reporting System (FARS; *n* = 27), Youth Risk Behavior Survey (YRBS; *n* = 17), and Behavioral Risk Factor Surveillance System (BRFSS; *n* = 15). The remaining data sources were used by 6–12 studies each: Treatment Episode Data Set (TEDS), National Incident‐Based Reporting System (NIBRS), Healthcare Cost and Utilization Project (HCUP), National College Health Assessment (NCHA), National Poison Data System (NPDS), Monitoring the Future (MTF), and National Longitudinal Survey of Youth (NLSY).

#### 5.3.6 Target population

**Figure 11:**
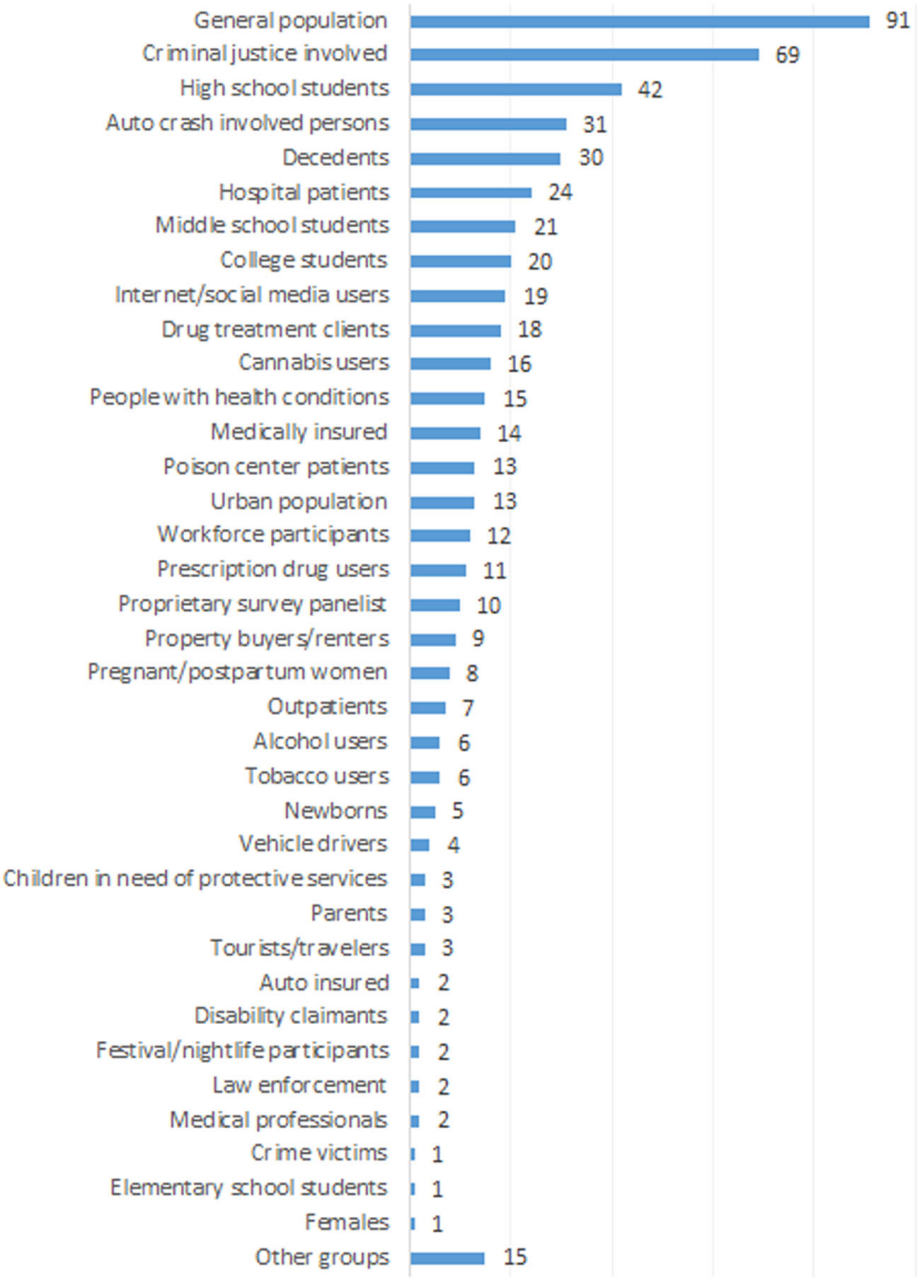
Number of primary studies by target population.

Figure 11 depicts 37 target populations coded for this EGM. We found the general population to be the most common reference group (*n* = 91), followed by criminal justice involved individuals (*n* = 69), high school students (*n* = 42), auto crash involved persons (*n* = 31), and decedents (*n* = 30). Overall, the diversity of target populations is indicative of the broad range of outcomes identified and researcher interest in understanding the indirect effects of cannabis policy (e.g., prenatal exposure among newborns and low birth weight).

**Figure 12:**
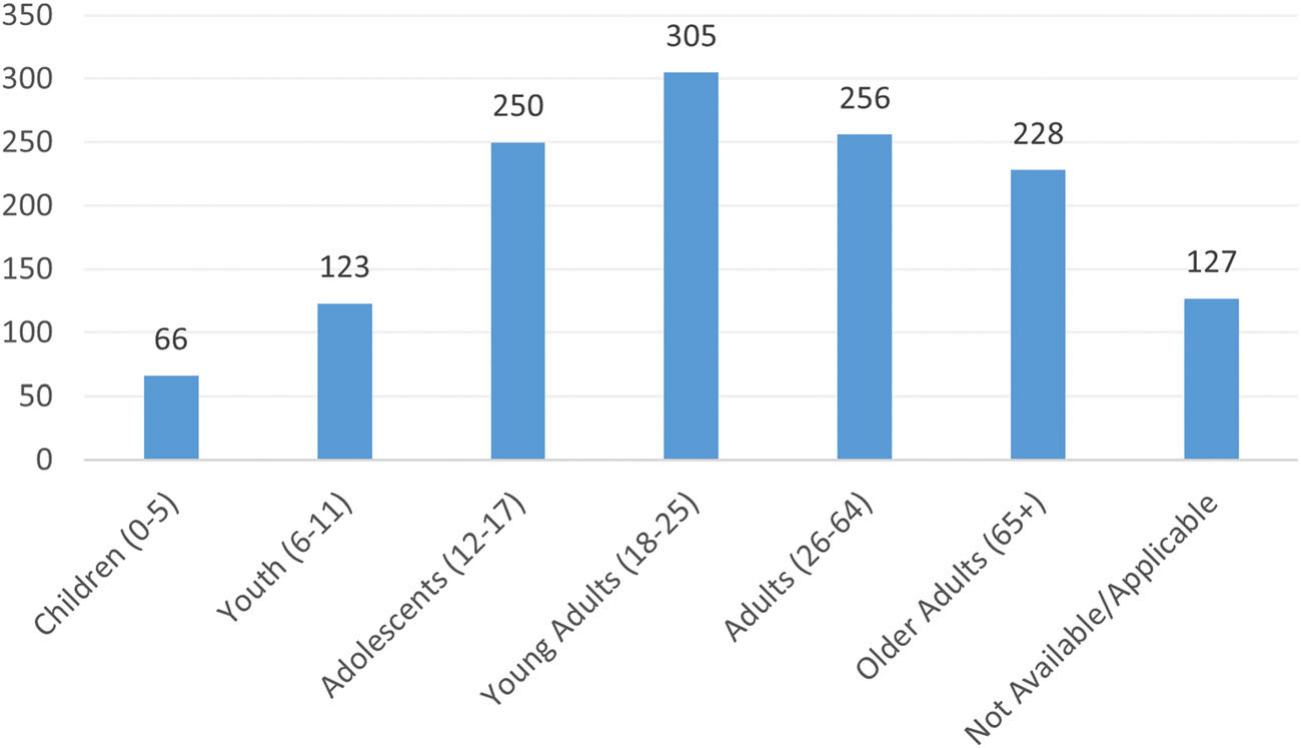
Number of primary studies by target population age group.

Figure 12 breaks down the target populations by age group. Most studies (*n* = 305) focused on the young adult (18–25) population, whereas small children (0–5) were studied least often (*n* = 66). For 127 studies, subject age was either not reported or not applicable.

#### 5.3.7 Publication type

Most primary studies in the EGM are peer‐reviewed journal articles (*n* = 298), followed by undergraduate or graduate dissertations and theses (*n* = 85), unpublished working papers (*n* = 34), and conference proceedings or abstracts (*n* = 21).

#### 5.3.8 Funding status

Most primary studies did not report funding status (*n* = 215). However, when this information was reported, more than four studies were funded (*n* = 183) for every unfunded study (*n* = 40).

#### 5.3.9 Research design and quality appraisal

**Figure 13:**
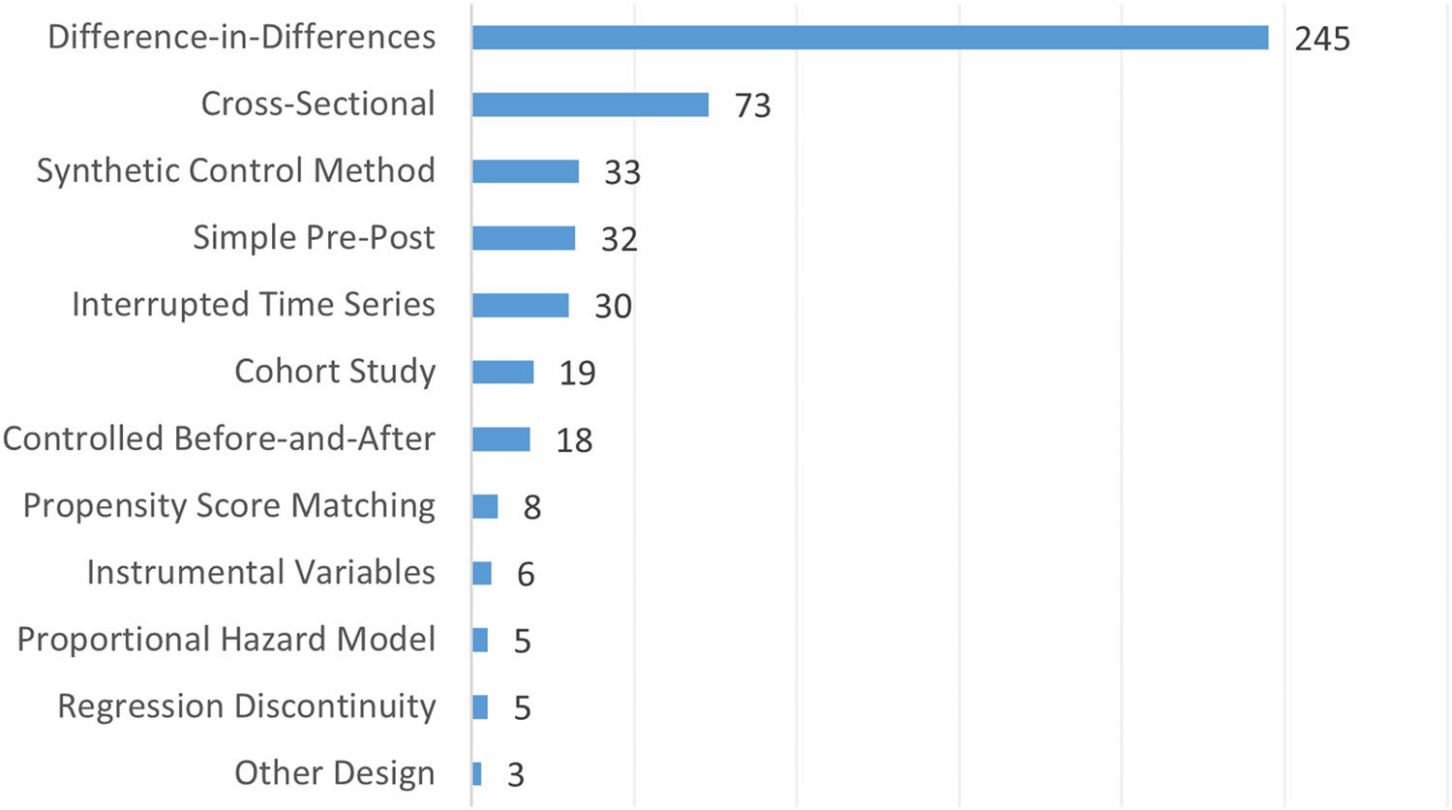
Number of primary studies by research design.

Figure 13 presents the number of primary studies by quasi‐experimental research design. Given its flexibility in estimating effects with staggered policy adoption across jurisdictions, difference‐in‐differences (DID) was the most frequently used design (*n* = 245) in the EGM. The ubiquity of the DID design meant it was often estimated in concert with supplementary designs. For instance, the synthetic control method was used in 33 studies, but DID was a supplemental method in 21 of these studies. After DID and SCM, the most common designs were cross‐sectional (*n* = 73) and simple pre‐post (*n* = 32), which are among the least rigorous from a causal analysis perspective. In contrast, the most methodologically sophisticated designs, including instrumental variables (*n* = 6) and regression discontinuity (*n* = 5), were rarely used in this literature.

The distribution of quality appraisals based on the SMS indicates that about one‐quarter of the studies in the EGM (*n* = 109) received a minimal quality appraisal and 10% (*n* = 44) attained a low rating. These lower‐rated studies do not support strong causal claims. Most studies in the EGM (*n* = 281) achieved a moderate quality appraisal, which is often considered the minimally acceptable rating for causal inference. Lastly, just a handful of studies (*n* = 11) earned a high quality appraisal. Although the SMS does not provide a comprehensive assessment of risk of bias, about two‐thirds (65.3%) of primary studies included in this review used designs that are causally robust.

### 5.4 Description of included systematic reviews

We documented seven completed and two ongoing systematic reviews in the EGM. The ongoing reviews, registered in PROSPERO, will investigate the effects of cannabis liberalization laws on health‐related outcomes (Busse, 2018) and youth attitudes, perceived risks, and cannabis‐related healthcare utilization (Stitchell, 2020). Both ongoing studies appear stalled, however, as the registered protocols have not been updated recently.

The seven completed systematic reviews are summarized in Table 6, including one rapid review (Ansari, 2020), three narrative reviews (Guttmannova, 2016; Leung, 2018; Vyas, 2018), and three reviews with meta‐analyses (Chihuri, 2019; Melchior, 2019; Sarvet, 2018). Five reviews focused on cannabis liberalization laws generally (including one that was part of a broader review of opioid interventions), whereas two targeted just MCLs. Three reviews addressed cannabis use and cannabis use disorders, three targeted opioid‐related harms, and one focused on alcohol‐related outcomes. Five reviews were delimited to the United States, one to both the United States and Canada, and one was not delimited geographically. The number of included studies ranged between 9 and 41.

**Table 6:** Summary of systematic reviews included in the EGM.

Review	Review type	Review focus	Setting and window	Included studies and quality appraisal^a^
Ansari et al. (2020)	Rapid review	Effects of systems‐level opioid policy interventions on opioid use, overdose, or death. MCLs and RCLs included among target interventions.	United States and Canada, January 1, 2014 through July 19, 2018	Included 9 relevant studies. AMSTAR 2 appraisal by domain: (1) Yes, (2) No, (3) Yes, (4) No, (5) Yes, (6) Yes, (7) No, (8) Yes, (9) No, (10) No, (11) N/A, (12) N/A, (13) No, (14) No, (15) N/A, (16) Yes. Overall confidence in results: Minimal.
Chihuri and Li (2019)	Meta‐analysis	Effects of cannabis liberalization laws on opioid overdose, mortality, and related health outcomes.	United States, through March 15, 2019	Included 16 studies. AMSTAR 2 appraisal by domain: (1) Yes, (2) No, (3) Yes, (4) Yes, (5) Yes, (6) Yes, (7) No, (8) Yes, (9) Partial Yes, (10) No, (11) Yes, (12), No, (13) No, (14) Yes, (15) Yes, (16) Yes. Overall confidence in results: Minimal.
Guttmannova et al. (2016)	Narrative review	Effects of cannabis liberalization laws, including decriminalization, on alcohol‐related outcomes.	United States, through 2015	Included 15 studies. AMSTAR 2 appraisal by domain: (1) Yes, (2) No, (3) Yes, (4) Partial Yes, (5) No, (6) No, (7) No, (8) No, (9) No, (10) No, (11) N/A, (12) N/A, (13) No, (14) No, (15) N/A, (16) Yes. Overall confidence in results: Minimal.
Leung et al. (2018)	Narrative review	Effects of cannabis liberalization laws on cannabis use and cannabis use disorder.	Destrée et al., 2018	Included 16 studies. AMSTAR 2 appraisal by domain: (1) Yes, (2) No, (3) Yes, (4) No, (5) No, (6) No, (7) No, (8) Partial Yes, (9) No, (10) Yes, (11) N/A, (12) N/A, (13) No, (14) No, (15) N/A, (16) Yes. Overall confidence in results: Minimal. Overall confidence in results: Minimal.
Melchior et al. (2019)	Meta‐analysis	Effects of cannabis liberalization laws on adolescent and young adult cannabis use.	Any country, through March 1, 2018	Included 41 studies. AMSTAR 2 appraisal by domain: (1) Yes, (2) Yes, (3) Yes, (4) Partial Yes, (5) Yes, (6) No, (7) No, (8) Yes, (9) Partial Yes, (10) No, (11) Yes, (12), No, (13) Yes, (14) Yes, (15) Yes, (16) Yes, Overall confidence in results: Low.
Sarvet et al. (2018)	Meta‐analysis	Effects of MCLs on adolescent cannabis use.	United States, through 2014	Included 21 studies (11 in meta‐analysis). AMSTAR 2 appraisal by domain: (1) Yes, (2) No, (3) Yes, (4) Partial Yes, (5) Yes, (6) No, (7) No, (8) Yes, (9) No, (10) No, (11) Yes, (12), No, (13) No, (14) Yes, (15) No, (16) Yes. Overall confidence in results: Low.
Vyas et al. (2018)	Narrative review	Effects of MCLs on prescription opioid use and opioid use disorder.	United States, 2010 through July 2017	Included 10 studies. AMSTAR 2 appraisal by domain: (1) Yes, (2) No, (3) Yes, (4) Partial Yes, (5) No, (6) No, (7) No, (8) Yes, (9) No, (10) No, (11) N/A, (12) N/A, (13) No, (14) No, (15) N/A, (16) No. Overall confidence in results: Minimal.

^a^ AMSTAR 2 quality appraisal domains: (1) PICO components, (2) Protocol, (3) Rationale for study inclusion, (4) Comprehensive search strategy, (5) Duplicate screening, (6) Duplicate coding, (7) List of excluded studies, (8) Description of included studies, (9) RoB assessment, (10) Source of funding, (11) Appropriate meta‐analysis, (12) Impact of RoB in individual studies, (13) Use RoB in interpreting results, (14) Address study heterogeneity, (15) Analyze publication bias, (16) Report conflict of interest.

**Figure 14:**
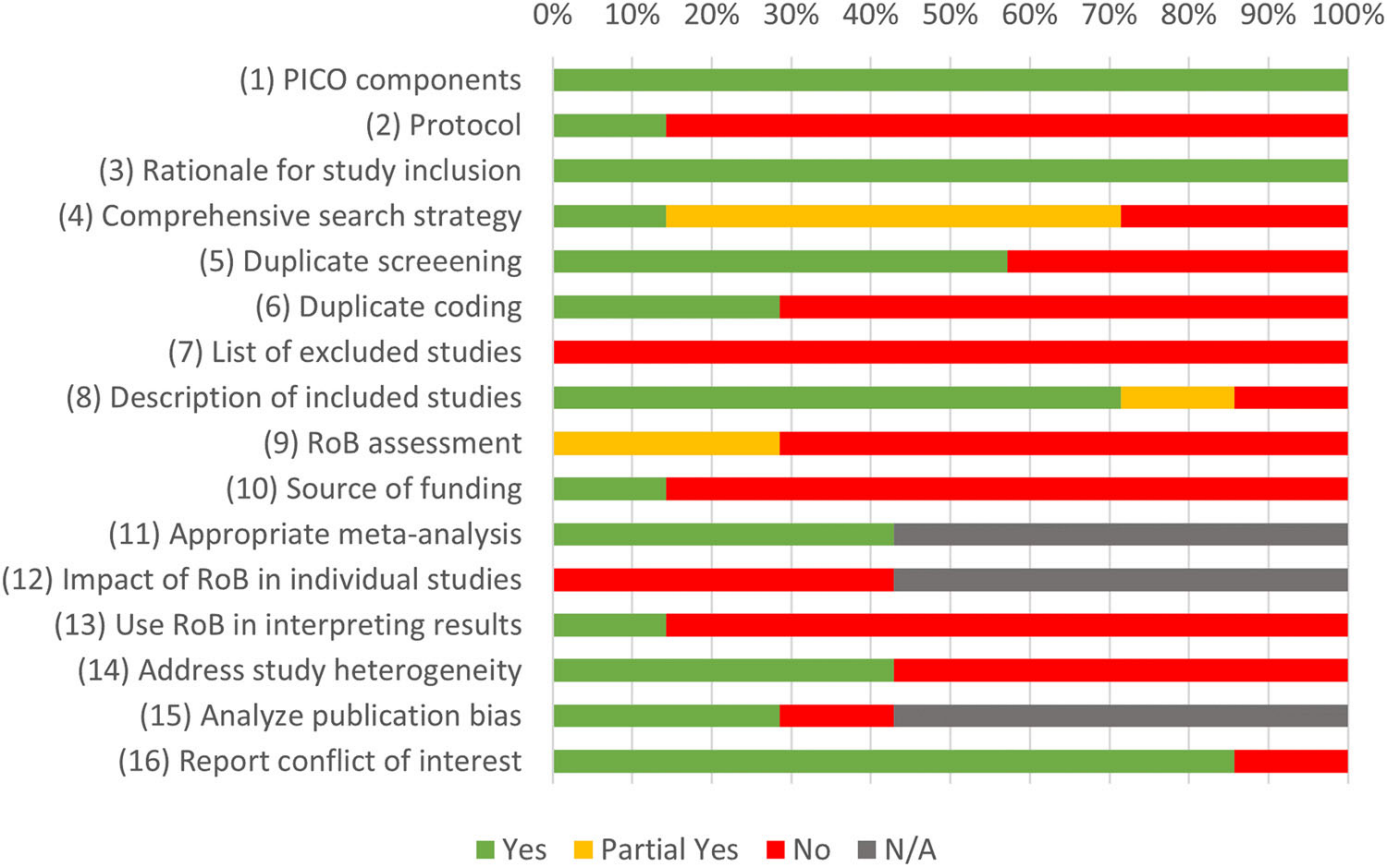
AMSTAR 2 assessments.

We critically appraised the seven completed systematic reviews using AMSTAR 2 (Shea, 2017). Table 6 reports (i) domain‐level scoring for each review and (ii) an overall confidence rating based on seven critical domains per AMSTAR 2 guidance. Five reviews were rated lowest as having minimal, and two were rated marginally better as having low confidence in the results. Notably, no reviews achieved a moderate or high rating. As shown in Figure 14, common review limitations include the lack of a prespecified research protocol, implementation of a limited search strategy (two reviews queried just a single database), single study screening or coding, failure to document the source of funding or list of excluded studies, and inadequate risk of bias or effect heterogeneity assessment. The AMSTAR 2 ratings highlight the shortcomings of existing reviews, which reinforces the need for updated and well‐implemented systematic reviews and meta‐analyses.

## 6 DISCUSSION

The objectives of this EGM were to develop a conceptual framework linking cannabis liberalization policies to relevant outcomes, descriptively summarize the empirical evidence, and identify areas of evidence concentration and gaps. This section contextualizes our results by reviewing patterns observed in the literature and presenting a holistic overview of the evidence base.

### 6.1 Areas of evidence concentration and evidence gaps

Virtually all (98.8%) cannabis liberalization research included in this EGM focused on the impacts of MCLs (*n* = 273) or RCLs (*n* = 241). The heaviest concentration of primary MCL and RCL studies targeted substance use generally (*n* = 129 and 103, respectively) and cannabis use specifically (*n* = 91 and 80, respectively). This clustering is also reflected in the seven completed systematic reviews in the EGM: all assessed substance use outcomes, and three specifically targeted cannabis use.

While primary studies of substance use predominated, a fair degree of evidence saturation emerged across other subdomains. Moderately high concentrations of MCL studies focused on crime and justice (*n* = 52), substance use disorders (*n* = 33), attitudes and perceptions (*n* = 28), and roadway safety (*n* = 28). Likewise, we documented moderately high concentrations of primary RCL studies in the areas of crime and justice (*n* = 39), attitudes and perceptions (*n* = 36), and roadway safety (*n* = 35). These are areas where ongoing and future systematic review efforts are warranted.

Smaller yet meaningful aggregations of evidence on the effects of MCLs were apparent across a handful of other subdomains, including mortality (*n* = 20), drug markets (*n* = 18), mental health (*n* = 14), and poisoning and overdose (*n* = 12). Similarly, for RCLs, we observed moderately low concentrations of evidence for substance use disorders (*n* = 19), drug markets (*n* = 16), business and economy (*n* = 15), poisoning and overdose (*n* = 12), and mortality (*n* = 11).

In addition to general‐law studies, the EGM investigated the impact of specific cannabis policy provisions. We observed, across both MCLs and RCLs, that most policy provision studies sought to understand the effects of legal sales through retail outlets or dispensaries. Overall, the evidence from 181 dispensary studies clustered predominately in four outcome subdomains: substance use (*n* = 74), crime and justice (*n* = 39), substance use disorders (*n* = 21), and roadway safety (*n* = 20). Similarly, a smaller aggregation of 61 MCL studies that examined the effects of home cultivation, patient registries, and/or patient qualifying conditions primarily investigated substance use (*n* = 29) and crime and justice (*n* = 13) outcomes.

The EGM also highlighted major gaps in the evidence. Notably, just ten primary studies investigated the effects of cannabis laws legalizing hemp production (IHLs), medicalizing CBD products (CBDLs), or decriminalizing cannabis cultivation (DCLs). This research gap can be explained in part by the later adoption, slower diffusion, and narrower scope of these laws. Major evidence gaps were also evident for more heavily researched MCLs and RCLs. Outcome subdomains for which the combined number of primary studies on MCLs and RCLs remained relatively sparse include demographic shifts (*n* = 9), policing activities (*n* = 9), healthcare and insurance impacts (*n* = 7), child and maternal health (*n* = 6), educational attainment (*n* = 6), and advertising and marketing exposures (*n* = 5). Moreover, evidence on MCLs and RCLs was slim to nonexistent across other key subdomains: sexual activity and other behaviors (*n* = 3), workplace safety (*n* = 2), and environmental impacts (*n* = 0). Still, even when there was a modicum of evidence on MCLs and RCLs across certain subdomains, the number of studies addressing specific outcomes was often slim. For instance, 17 MCL/RCL studies assessed physical health concerns overall, but specific physical health maladies were investigated by just one (i.e., sexually transmitted diseases, inflammatory conditions, sleep disorders, and spasticity) or two (i.e., chronic pain, injury/trauma, and respiratory health) primary studies.

### 6.2 Quality of the evidence

Overall, most primary studies (65%) in the EGM were rated moderate to high quality based on the Maryland Scientific Methods Scale (Madaleno, 2016; Sherman, 2002). Studies appraised moderate to high quality are generally considered more robust for making causal claims than studies rated minimal or low quality.

Among the nine systematic reviews included in the EGM, five were rated minimal quality according to AMSTAR 2 (Shea, 2017), two were rated low quality, and two were not rated as they are technically still in progress. Notably, no systematic reviews were rated as being of moderate or high quality, suggesting a critical need for more robust syntheses of existing evidence.

### 6.3 Limitations of the EGM

The EGM involved an extensive search for peer‐reviewed and gray literature, screening nearly 17,000 citations. Despite this, there are several limitations to our search process. First, we included only English language studies in the EGM, which may have limited the inclusion of eligible studies from other countries. Second, we used artificial intelligence screening tools to increase screening efficiency, but it is possible that some eligible studies that we did not manually screen were erroneously excluded. Third, this is a policy area with research being published at a rapid pace, so the EGM will not capture the most recent evidence. After rerunning our original search for the recent period August 10, 2020 to August 10, 2022, we retrieved an additional 4,132 potentially relevant citations to screen. The EGM will therefore need to be updated regularly.

Although we used the SMS to assess the robustness of study designs, we did not fully assess the implementation quality of these designs. For instance, not all difference‐in‐differences studies included in the EGM are equally robust. Future evidence syntheses should enhance quality appraisal. Finally, our coding of study outcomes and target populations was purposely open‐ended to accommodate the interdisciplinary breadth of cannabis liberalization research. Accordingly, our categorization of these outcomes and target populations, although embedded within a clear conceptual framework, was not thoroughly prespecified in our protocol.

## 7 AUTHORS’ CONCLUSIONS

Across the globe, the international status quo of strict cannabis prohibition is rapidly ceding to a range of cannabis policy reforms aimed at liberalizing access to cannabis for medical and recreational purposes. This EGM provides a comprehensive summary of existing research examining the public health, community safety, and socioeconomic implications of these reforms. It highlights areas of evidence concentrations and evidence gaps to inform future research needs, including both primary studies and systematic reviews.

### 7.1 Implications for research and policy

The modal primary study in this EGM investigates the effects of MCLs or RCLs on substance use, namely cannabis use. In our conceptual framework, substance use is a key intermediate outcome, but more primary and systematic research is needed to better understand the effects of cannabis liberalization laws on longer‐term—and arguably more salient—health, safety, and socioeconomic outcomes. This EGM, as well as other recent conceptualizations of cannabis policy outcomes (e.g., Campeny, 2020; Daldegan‐Bueno, 2022; Lake, 2019), can assist in prioritizing such a research program. Moreover, future research might productively employ more nuanced cannabis use measures (e.g., frequency and modes of use) as mediators in research on causal mechanisms influencing ultimate outcomes.

This EGM has also highlighted the heterogeneous nature of cannabis liberalization laws. We observed a critical need for research addressing industrial hemp production, medical CBD products, and decriminalized cannabis cultivation. Most research in this policy space focused on MCL and RCL general‐law effects. To the extent that specific policy provisions were investigated, studies predominantly assessed the effects of cannabis dispensaries. Future research should prioritize understanding how other key policy provisions impact relevant outcomes. Reliance on standardized policy databases such as the Prescription Drug Abuse Monitoring System (PDAPS) and Alcohol Policy Information System (APIS) can facilitate this effort.

The existing body of research on cannabis liberalization policy is derived primarily from the United States. More research is desperately needed from other countries to diversify our understanding of these laws, as current evidence may not be generalizable to other settings.

Methodologically, cannabis policy research would greatly benefit from improvements in measurement and design aimed at enhancing causal validity and inference (Hunt, 2015). Many studies of crime and driving consequences, for instance, rely on administrative databases, such as the Uniform Crime Reports (UCR) and Fatality Analysis Reporting System (FARS), which do a poor job of measuring cannabis‐specific outcomes. As a result, researchers commonly default to operationalizing general arrest and crash outcomes instead. However, researchers are overcoming these deficiencies by using novel data systems and more advanced methods. For example, Firth et al. (Firth, 2019) used the National Incident‐Based Reporting System (NIBRS), which has certain advantages over UCR, to investigate post‐legalization changes in racial disparities in cannabis arrests in Washington state (also see Doonan, 2020). As another example, Sevigny (Sevigny, 2018) accounted for missing data in FARS by using multiple imputation to more reliably measure cannabis positivity among drivers involved in fatal crashes.

From a design perspective, we noted that most research in this area employs a difference‐in‐differences (DID) design, estimated primarily by way of two‐way fixed‐effects (TWFE). Recent advances in the econometrics of DID, however, call into serious question the validity of TWFE when policy adoption is staggered and effects are heterogeneous (Goodman‐Bacon, 2021; Roth, 2022). Since these features are common in cannabis policy research, there is a critical need for replication using alternative DID estimators that counter the potential bias inherent in the canonical TWFE implementation (e.g., Callaway, 2021; Sant'Anna, 2020; Sun, 2021). The implications of these new methods are far‐reaching, and serious scholars in the field should take note of these and related methodological innovations (e.g., Ben‐Michael, 2022; de Chaisemartin, 2020) in designing future research studies.

Despite the global proliferation of cannabis liberalization policies and the associated accumulation of empirical research, especially over the last decade, clear implications of this massive social policy experiment remain elusive. Accordingly, this EGM highlights a critical need for more robust primary studies and systematic reviews on a broader range of salient outcomes.

## CONTRIBUTIONS OF AUTHORS

1

**Eric L. Sevigny**: Conceptualization; methodology; software; validation; formal analysis; investigation; resources; data curation; writing—original draft; visualization; supervision; project administration; funding acquisition. **Danye N. Medhin**: Software; validation; investigation; data curation; writing—review and editing. **Jared Greathouse**: Software; validation; formal analysis; investigation; data curation; writing—review and editing; visualization.

## DECLARATIONS OF INTEREST

The authors have no vested interest in the outcomes of this EGM, nor any incentive to represent findings in a biased manner. Sevigny has published primary research in this policy area, including two studies included in this EGM. He was not involved in the coding of these studies.

## PLANS FOR UPDATING THE EGM

Once published, we plan to update the EGM on a regular basis, contingent upon the availability of external funding. At present, we have rerun our systematic literature search for the period August 10, 2020 to August 10, 2022 and retrieved an additional 4132 unduplicated references for further screening and coding. The lead author will be responsible for updating the EGM.

## DIFFERENCES BETWEEN PROTOCOL AND REVIEW

The protocol for this EGM was previously published (Sevigny et al., 2021). No deviations were made to study eligibility criteria or the literature search strategy. We amended the conceptual framework depicted in Figure 1 to better highlight the direct effect of legal practices on final outcomes while de‐emphasizing potential feedback between intermediate factors, which was ultimately of secondary interest for this review. In the final review, we also revised our coding guide in planned and unplanned ways. Given that our purview was explicitly open‐ended with respect to coding outcomes and target populations, we revised our coding scheme to reflect the specific categories and groups encountered in the review. However, we amended our coding of interventions. In the protocol, we listed contingent provisions under various categories (e.g., user authorization provisions, commercial supply provisions) without being explicitly nested under each parent law. In the final review, we coded specific provisions nested by policy type. Since available studies of CBDLs, IHLs, and DCLs reported only general‐law effects, we simply coded the high‐level policies. For MCLs and RCLs, we coded provisions that were relevant to each specific law. In adopting this strategy, we aggregated, dropped, or added certain provisions to simplify the coding. For instance, we merged “advertising regulations” and “product display regulations” described in the protocol into a single “product and marketing regulations” category in the final review. Similarly, we dropped provisions for “taxes” and “banking regulations” as these were never investigated in the literature and rarely, if ever, included in statutory language. And, we added codes for unanticipated policy operationalizations, namely “policy indexes” that combine multiple policy provisions into a single measure. Lastly, we did not anticipate the use of AI during study screening in the protocol because we were unaware of these tools during protocol development. However, after DistillerSR launched these tools on its platform, we decided to learn about them and use them to screen studies when conducting the final review.

## SOURCES OF SUPPORT

Internal sources

- No sources of support provided

External sources

• National Institute on Drug Abuse, USA

NIH Award No.: R03DA046806

## Supporting information

Supporting information.
