# Supplementary material for: Health, safety, and socioeconomic impacts of cannabis liberalization laws: An evidence and gap map
Source: Campbell Syst Rev. 2023 Oct 30;19(4):e1362. doi: 10.1002/cl2.1362 (PMC10616541; doi:10.1002/cl2.1362)
Supplement: Supplementary file 1 — Supporting information. [file CL2-19-e1362-s001.docx]

# Appendices

## 1 Literature search strategy and terms

This appendix provides the exact search strategy and criteria used for all academic databases and gray literature sources.

**(1) Academic Search Complete (EBSCO)**

August 13, 2020; 1972-2020; Publication types: Excludes magazines, trade publications, newspapers, book reviews; English. Fields searched: title, abstract, keyword, subject.

(TI (cannabis OR marijuana OR hemp) OR AB (cannabis OR marijuana OR hemp) OR KW (cannabis OR marijuana OR hemp) OR SU (“cannabis” OR “cannabis edibles” OR “hemp” OR “marijuana” OR “medical marijuana”)) **AND** (TI (commercializ* OR “social club*” OR collective OR cooperative OR cultivat* OR decrim* OR dispensar* OR grow* OR industr* OR law* OR legal* OR legislat* OR liberaliz* OR medical* OR polic* OR program* OR recreation* OR regulat* OR retail*) OR AB (commercializ* OR “social club*” OR collective OR cooperative OR cultivat* OR decrim* OR dispensar* OR grow* OR industr* OR law* OR legal* OR legislat* OR liberaliz* OR medical* OR polic* OR program* OR recreation* OR regulat* OR retail*) OR KW (commercializ* OR “social club*” OR collective OR cooperative OR cultivat* OR decrim* OR dispensar* OR grow* OR industr* OR law* OR legal* OR legislat* OR liberaliz* OR medical* OR polic* OR program* OR recreation* OR regulat* OR retail*) OR SU (“commercialization” OR “drug control” OR “drug laws & regulations” OR “drug legalization” OR “government policy” OR “government regulation” OR “government regulations” OR “law” & “legalization” OR “legislation” OR “legislation, drug” OR “legislation, medical” OR “liberalization” OR “marijuana dispensaries” OR “marijuana growing” OR “marijuana industry” OR “marijuana laws” OR “marijuana legalization” OR “medical marijuana -- law & legislation” OR “medicalization” OR “public policy” OR “public policy (law)” OR “regulations” OR “retail stores” OR “state laws” OR “state regulation”)) **AND** (TI (analysis OR “before-and-after” OR causal OR counterfactual OR “difference-in-difference*” OR experiment* OR “fixed effect*” OR “instrumental variable*” OR longitudinal OR matching OR meta-analy* OR model* OR “panel data” OR “propensity score” OR “propensity match*” OR quasi-experiment* OR regress* OR “regression discontinuity” OR “repeated measures” OR RCT OR “synthetic control” OR “systematic review” OR “time-series”) OR AB (analysis OR “before-and-after” OR causal OR counterfactual OR “difference-in-difference*” OR experiment* OR “fixed effect*” OR “instrumental variable*” OR longitudinal OR matching OR meta-analy* OR model* OR “panel data” OR “propensity score” OR “propensity match*” OR quasi-experiment* OR regress* OR “regression discontinuity” OR “repeated measures” OR RCT OR “synthetic control” OR “systematic review” OR “time-series”) OR KW (analysis OR “before-and-after” OR causal OR counterfactual OR “difference-in-difference*” OR experiment* OR “fixed effect*” OR “instrumental variable*” OR longitudinal OR matching OR meta-analy* OR model* OR “panel data” OR “propensity score” OR “propensity match*” OR quasi-experiment* OR regress* OR “regression discontinuity” OR “repeated measures” OR RCT OR “synthetic control” OR “systematic review” OR “time-series”) OR SU (“causal modeling” OR “causal models” OR “controlled before-after studies” OR “controlled before and after studies” OR “data analysis” OR “data analysis, statistical” OR “econometric models” OR “fixed effects model” OR “granger causality test” OR “instrumental variables (statistics)” OR “instrumental variable estimation” OR “interrupted time series analysis” OR “longitudinal method” OR “longitudinal studies” OR “meta-analysis” OR “panel analysis” OR “panel data” OR “policy analysis” OR “population-based case control” OR “pretests posttests” OR “pretest-posttest design” OR “propensity score” OR “propensity score matching” OR “statistical analysis” OR “statistical matching” OR “quantitative research” OR “quasi-experimental studies” OR “regression” OR “regression analysis” OR “regression discontinuity design” OR “repeated measures” OR “repeated measures design” OR “time series” OR “time series analysis”))

**(2) APA PsycInfo (EBSCO)**

August 13, 2020; 1970-2020; English. Fields searched: title, abstract, keyword, subject.

(TI (cannabis OR marijuana OR hemp) OR AB (cannabis OR marijuana OR hemp) OR KW (cannabis OR marijuana OR hemp) OR SU (“cannabis” OR “cannabis edibles” OR “hemp” OR “marijuana” OR “medical marijuana”)) **AND** (TI (commercializ* OR “social club*” OR collective OR cooperative OR cultivat* OR decrim* OR dispensar* OR grow* OR industr* OR law* OR legal* OR legislat* OR liberaliz* OR medical* OR polic* OR program* OR recreation* OR regulat* OR retail*) OR AB (commercializ* OR “social club*” OR collective OR cooperative OR cultivat* OR decrim* OR dispensar* OR grow* OR industr* OR law* OR legal* OR legislat* OR liberaliz* OR medical* OR polic* OR program* OR recreation* OR regulat* OR retail*) OR KW (commercializ* OR “social club*” OR collective OR cooperative OR cultivat* OR decrim* OR dispensar* OR grow* OR industr* OR law* OR legal* OR legislat* OR liberaliz* OR medical* OR polic* OR program* OR recreation* OR regulat* OR retail*) OR SU (“commercialization” OR “drug control” OR “drug laws & regulations” OR “drug legalization” OR “government policy” OR “government regulation” OR “government regulations” OR “law” & “legalization” OR “legislation” OR “legislation, drug” OR “legislation, medical” OR “liberalization” OR “marijuana dispensaries” OR “marijuana growing” OR “marijuana industry” OR “marijuana laws” OR “marijuana legalization” OR “medical marijuana -- law & legislation” OR “medicalization” OR “public policy” OR “public policy (law)” OR “regulations” OR “retail stores” OR “state laws” OR “state regulation”)) **AND** (TI (analysis OR “before-and-after” OR causal OR counterfactual OR “difference-in-difference*” OR experiment* OR “fixed effect*” OR “instrumental variable*” OR longitudinal OR matching OR meta-analy* OR model* OR “panel data” OR “propensity score” OR “propensity match*” OR quasi-experiment* OR regress* OR “regression discontinuity” OR “repeated measures” OR RCT OR “synthetic control” OR “systematic review” OR “time-series”) OR AB (analysis OR “before-and-after” OR causal OR counterfactual OR “difference-in-difference*” OR experiment* OR “fixed effect*” OR “instrumental variable*” OR longitudinal OR matching OR meta-analy* OR model* OR “panel data” OR “propensity score” OR “propensity match*” OR quasi-experiment* OR regress* OR “regression discontinuity” OR “repeated measures” OR RCT OR “synthetic control” OR “systematic review” OR “time-series”) OR KW (analysis OR “before-and-after” OR causal OR counterfactual OR “difference-in-difference*” OR experiment* OR “fixed effect*” OR “instrumental variable*” OR longitudinal OR matching OR meta-analy* OR model* OR “panel data” OR “propensity score” OR “propensity match*” OR quasi-experiment* OR regress* OR “regression discontinuity” OR “repeated measures” OR RCT OR “synthetic control” OR “systematic review” OR “time-series”) OR SU (“causal modeling” OR “causal models” OR “controlled before-after studies” OR “controlled before and after studies” OR “data analysis” OR “data analysis, statistical” OR “econometric models” OR “fixed effects model” OR “granger causality test” OR “instrumental variables (statistics)” OR “instrumental variable estimation” OR “interrupted time series analysis” OR “longitudinal method” OR “longitudinal studies” OR “meta-analysis” OR “panel analysis” OR “panel data” OR “policy analysis” OR “population-based case control” OR “pretests posttests” OR “pretest-posttest design” OR “propensity score” OR “propensity score matching” OR “statistical analysis” OR “statistical matching” OR “quantitative research” OR “quasi-experimental studies” OR “regression” OR “regression analysis” OR “regression discontinuity design” OR “repeated measures” OR “repeated measures design” OR “time series” OR “time series analysis”))

**(3) Aplied Social Sciences Index & Abstracts (ASSIA) (ProQuest)**

August 13, 2020; 1973-2020; Publication types: Excludes commentary, editorial, general information, correspondence, letter to the editor, news; English. Fields searched: title, abstract, keyword, subject.

(ti(cannabis OR marijuana OR hemp) OR ab(cannabis OR marijuana OR hemp) OR if(cannabis OR marijuana OR hemp) OR su(“cannabis” OR “cannabis edibles” OR “hemp” OR “marijuana” OR “medical marijuana”)) **AND** (ti(commercializ* OR “social club*” OR collective OR cooperative OR cultivat* OR decrim* OR dispensar* OR grow* OR industr* OR law* OR legal* OR legislat* OR liberaliz* OR medical* OR polic* OR program* OR recreation* OR regulat* OR retail*) OR ab(commercializ* OR “social club*” OR collective OR cooperative OR cultivat* OR decrim* OR dispensar* OR grow* OR industr* OR law* OR legal* OR legislat* OR liberaliz* OR medical* OR polic* OR program* OR recreation* OR regulat* OR retail*) OR if(commercializ* OR “social club*” OR collective OR cooperative OR cultivat* OR decrim* OR dispensar* OR grow* OR industr* OR law* OR legal* OR legislat* OR liberaliz* OR medical* OR polic* OR program* OR recreation* OR regulat* OR retail*) OR su(“commercialization” OR “drug control” OR “drug laws & regulations” OR “drug legalization” OR “government policy” OR “government regulation” OR “government regulations” OR “law” & “legalization” OR “legislation” OR “legislation, drug” OR “legislation, medical” OR “liberalization” OR “marijuana dispensaries” OR “marijuana growing” OR “marijuana industry” OR “marijuana laws” OR “marijuana legalization” OR “medical marijuana -- law & legislation” OR “medicalization” OR “public policy” OR “public policy (law)” OR “regulations” OR “retail stores” OR “state laws” OR “state regulation”)) **AND** (ti(analysis OR “before-and-after” OR causal OR counterfactual OR “difference-in-difference*” OR experiment* OR “fixed effect*” OR “instrumental variable*” OR longitudinal OR matching OR meta-analy* OR model* OR “panel data” OR “propensity score” OR “propensity match*” OR quasi-experiment* OR regress* OR “regression discontinuity” OR “repeated measures” OR RCT OR “synthetic control” OR “systematic review” OR “time-series”) OR ab(analysis OR “before-and-after” OR causal OR counterfactual OR “difference-in-difference*” OR experiment* OR “fixed effect*” OR “instrumental variable*” OR longitudinal OR matching OR meta-analy* OR model* OR “panel data” OR “propensity score” OR “propensity match*” OR quasi-experiment* OR regress* OR “regression discontinuity” OR “repeated measures” OR RCT OR “synthetic control” OR “systematic review” OR “time-series”) OR if(analysis OR “before-and-after” OR causal OR counterfactual OR “difference-in-difference*” OR experiment* OR “fixed effect*” OR “instrumental variable*” OR longitudinal OR matching OR meta-analy* OR model* OR “panel data” OR “propensity score” OR “propensity match*” OR quasi-experiment* OR regress* OR “regression discontinuity” OR “repeated measures” OR RCT OR “synthetic control” OR “systematic review” OR “time-series”) OR su(“causal modeling” OR “causal models” OR “controlled before-after studies” OR “controlled before and after studies” OR “data analysis” OR “data analysis, statistical” OR “econometric models” OR “fixed effects model” OR “granger causality test” OR “instrumental variables (statistics)” OR “instrumental variable estimation” OR “interrupted time series analysis” OR “longitudinal method” OR “longitudinal studies” OR “meta-analysis” OR “panel analysis” OR “panel data” OR “policy analysis” OR “population-based case control” OR “pretests posttests” OR “pretest-posttest design” OR “propensity score” OR “propensity score matching” OR “statistical analysis” OR “statistical matching” OR “quantitative research” OR “quasi-experimental studies” OR “regression” OR “regression analysis” OR “regression discontinuity design” OR “repeated measures” OR “repeated measures design” OR “time series” OR “time series analysis”))

**(4) Campbell Library (Campbell Collaboration)**

August 13, 2020; Fields searched: title, keyword.

cannabis OR marijuana OR hemp

**(5) CINAHL Plus (EBSCO)**

August 13, 2020; 1981-2020; Publication types: Excludes magazines, CEUs; English. Fields searched: title, abstract, subject.

(TI (cannabis OR marijuana OR hemp) OR AB (cannabis OR marijuana OR hemp) OR SU (“cannabis” OR “cannabis edibles” OR “hemp” OR “marijuana” OR “medical marijuana”)) **AND** (TI (commercializ* OR “social club*” OR collective OR cooperative OR cultivat* OR decrim* OR dispensar* OR grow* OR industr* OR law* OR legal* OR legislat* OR liberaliz* OR medical* OR polic* OR program* OR recreation* OR regulat* OR retail*) OR AB (commercializ* OR “social club*” OR collective OR cooperative OR cultivat* OR decrim* OR dispensar* OR grow* OR industr* OR law* OR legal* OR legislat* OR liberaliz* OR medical* OR polic* OR program* OR recreation* OR regulat* OR retail*) OR SU (“commercialization” OR “drug control” OR “drug laws & regulations” OR “drug legalization” OR “government policy” OR “government regulation” OR “government regulations” OR “law” & “legalization” OR “legislation” OR “legislation, drug” OR “legislation, medical” OR “liberalization” OR “marijuana dispensaries” OR “marijuana growing” OR “marijuana industry” OR “marijuana laws” OR “marijuana legalization” OR “medical marijuana -- law & legislation” OR “medicalization” OR “public policy” OR “public policy (law)” OR “regulations” OR “retail stores” OR “state laws” OR “state regulation”)) **AND** (TI (analysis OR “before-and-after” OR causal OR counterfactual OR “difference-in-difference*” OR experiment* OR “fixed effect*” OR “instrumental variable*” OR longitudinal OR matching OR meta-analy* OR model* OR “panel data” OR “propensity score” OR “propensity match*” OR quasi-experiment* OR regress* OR “regression discontinuity” OR “repeated measures” OR RCT OR “synthetic control” OR “systematic review” OR “time-series”) OR AB (analysis OR “before-and-after” OR causal OR counterfactual OR “difference-in-difference*” OR experiment* OR “fixed effect*” OR “instrumental variable*” OR longitudinal OR matching OR meta-analy* OR model* OR “panel data” OR “propensity score” OR “propensity match*” OR quasi-experiment* OR regress* OR “regression discontinuity” OR “repeated measures” OR RCT OR “synthetic control” OR “systematic review” OR “time-series”) OR SU (“causal modeling” OR “causal models” OR “controlled before-after studies” OR “controlled before and after studies” OR “data analysis” OR “data analysis, statistical” OR “econometric models” OR “fixed effects model” OR “granger causality test” OR “instrumental variables (statistics)” OR “instrumental variable estimation” OR “interrupted time series analysis” OR “longitudinal method” OR “longitudinal studies” OR “meta-analysis” OR “panel analysis” OR “panel data” OR “policy analysis” OR “population-based case control” OR “pretests posttests” OR “pretest-posttest design” OR “propensity score” OR “propensity score matching” OR “statistical analysis” OR “statistical matching” OR “quantitative research” OR “quasi-experimental studies” OR “regression” OR “regression analysis” OR “regression discontinuity design” OR “repeated measures” OR “repeated measures design” OR “time series” OR “time series analysis”))

**(6) Cochrane Library (Wiley)**

August 13, 2020; Fields searched: title, abstract, keyword.

cannabis OR marijuana OR hemp

**(7) Criminal Justice Abstracts (EBSCO)**

August 13, 2020; 1970-2020; Publication types: Excludes magazines; English. Fields searched: title, abstract, keyword, subject.

(TI (cannabis OR marijuana OR hemp) OR AB (cannabis OR marijuana OR hemp) OR KW (cannabis OR marijuana OR hemp) OR SU (“cannabis” OR “cannabis edibles” OR “hemp” OR “marijuana” OR “medical marijuana”)) **AND** (TI (commercializ* OR “social club*” OR collective OR cooperative OR cultivat* OR decrim* OR dispensar* OR grow* OR industr* OR law* OR legal* OR legislat* OR liberaliz* OR medical* OR polic* OR program* OR recreation* OR regulat* OR retail*) OR AB (commercializ* OR “social club*” OR collective OR cooperative OR cultivat* OR decrim* OR dispensar* OR grow* OR industr* OR law* OR legal* OR legislat* OR liberaliz* OR medical* OR polic* OR program* OR recreation* OR regulat* OR retail*) OR KW (commercializ* OR “social club*” OR collective OR cooperative OR cultivat* OR decrim* OR dispensar* OR grow* OR industr* OR law* OR legal* OR legislat* OR liberaliz* OR medical* OR polic* OR program* OR recreation* OR regulat* OR retail*) OR SU (“commercialization” OR “drug control” OR “drug laws & regulations” OR “drug legalization” OR “government policy” OR “government regulation” OR “government regulations” OR “law” & “legalization” OR “legislation” OR “legislation, drug” OR “legislation, medical” OR “liberalization” OR “marijuana dispensaries” OR “marijuana growing” OR “marijuana industry” OR “marijuana laws” OR “marijuana legalization” OR “medical marijuana -- law & legislation” OR “medicalization” OR “public policy” OR “public policy (law)” OR “regulations” OR “retail stores” OR “state laws” OR “state regulation”)) **AND** (TI (analysis OR “before-and-after” OR causal OR counterfactual OR “difference-in-difference*” OR experiment* OR “fixed effect*” OR “instrumental variable*” OR longitudinal OR matching OR meta-analy* OR model* OR “panel data” OR “propensity score” OR “propensity match*” OR quasi-experiment* OR regress* OR “regression discontinuity” OR “repeated measures” OR RCT OR “synthetic control” OR “systematic review” OR “time-series”) OR AB (analysis OR “before-and-after” OR causal OR counterfactual OR “difference-in-difference*” OR experiment* OR “fixed effect*” OR “instrumental variable*” OR longitudinal OR matching OR meta-analy* OR model* OR “panel data” OR “propensity score” OR “propensity match*” OR quasi-experiment* OR regress* OR “regression discontinuity” OR “repeated measures” OR RCT OR “synthetic control” OR “systematic review” OR “time-series”) OR KW (analysis OR “before-and-after” OR causal OR counterfactual OR “difference-in-difference*” OR experiment* OR “fixed effect*” OR “instrumental variable*” OR longitudinal OR matching OR meta-analy* OR model* OR “panel data” OR “propensity score” OR “propensity match*” OR quasi-experiment* OR regress* OR “regression discontinuity” OR “repeated measures” OR RCT OR “synthetic control” OR “systematic review” OR “time-series”) OR SU (“causal modeling” OR “causal models” OR “controlled before-after studies” OR “controlled before and after studies” OR “data analysis” OR “data analysis, statistical” OR “econometric models” OR “fixed effects model” OR “granger causality test” OR “instrumental variables (statistics)” OR “instrumental variable estimation” OR “interrupted time series analysis” OR “longitudinal method” OR “longitudinal studies” OR “meta-analysis” OR “panel analysis” OR “panel data” OR “policy analysis” OR “population-based case control” OR “pretests posttests” OR “pretest-posttest design” OR “propensity score” OR “propensity score matching” OR “statistical analysis” OR “statistical matching” OR “quantitative research” OR “quasi-experimental studies” OR “regression” OR “regression analysis” OR “regression discontinuity design” OR “repeated measures” OR “repeated measures design” OR “time series” OR “time series analysis”))

**(8) Criminal Justice Database (ProQuest)**

August 13, 2020; 1981-2020; Publication types: Excludes magazines, trade journals; English. Fields searched: title, abstract, subject.

(ti(cannabis OR marijuana OR hemp) OR ab(cannabis OR marijuana OR hemp) OR su(“cannabis” OR “cannabis edibles” OR “hemp” OR “marijuana” OR “medical marijuana”)) **AND** (ti(commercializ* OR “social club*” OR collective OR cooperative OR cultivat* OR decrim* OR dispensar* OR grow* OR industr* OR law* OR legal* OR legislat* OR liberaliz* OR medical* OR polic* OR program* OR recreation* OR regulat* OR retail*) OR ab(commercializ* OR “social club*” OR collective OR cooperative OR cultivat* OR decrim* OR dispensar* OR grow* OR industr* OR law* OR legal* OR legislat* OR liberaliz* OR medical* OR polic* OR program* OR recreation* OR regulat* OR retail*) OR su(“commercialization” OR “drug control” OR “drug laws & regulations” OR “drug legalization” OR “government policy” OR “government regulation” OR “government regulations” OR “law” & “legalization” OR “legislation” OR “legislation, drug” OR “legislation, medical” OR “liberalization” OR “marijuana dispensaries” OR “marijuana growing” OR “marijuana industry” OR “marijuana laws” OR “marijuana legalization” OR “medical marijuana -- law & legislation” OR “medicalization” OR “public policy” OR “public policy (law)” OR “regulations” OR “retail stores” OR “state laws” OR “state regulation”)) **AND** (ti(analysis OR “before-and-after” OR causal OR counterfactual OR “difference-in-difference*” OR experiment* OR “fixed effect*” OR “instrumental variable*” OR longitudinal OR matching OR meta-analy* OR model* OR “panel data” OR “propensity score” OR “propensity match*” OR quasi-experiment* OR regress* OR “regression discontinuity” OR “repeated measures” OR RCT OR “synthetic control” OR “systematic review” OR “time-series”) OR ab(analysis OR “before-and-after” OR causal OR counterfactual OR “difference-in-difference*” OR experiment* OR “fixed effect*” OR “instrumental variable*” OR longitudinal OR matching OR meta-analy* OR model* OR “panel data” OR “propensity score” OR “propensity match*” OR quasi-experiment* OR regress* OR “regression discontinuity” OR “repeated measures” OR RCT OR “synthetic control” OR “systematic review” OR “time-series”) OR su(“causal modeling” OR “causal models” OR “controlled before-after studies” OR “controlled before and after studies” OR “data analysis” OR “data analysis, statistical” OR “econometric models” OR “fixed effects model” OR “granger causality test” OR “instrumental variables (statistics)” OR “instrumental variable estimation” OR “interrupted time series analysis” OR “longitudinal method” OR “longitudinal studies” OR “meta-analysis” OR “panel analysis” OR “panel data” OR “policy analysis” OR “population-based case control” OR “pretests posttests” OR “pretest-posttest design” OR “propensity score” OR “propensity score matching” OR “statistical analysis” OR “statistical matching” OR “quantitative research” OR “quasi-experimental studies” OR “regression” OR “regression analysis” OR “regression discontinuity design” OR “repeated measures” OR “repeated measures design” OR “time series” OR “time series analysis”))

**(9) Dissertations & Theses A&I (ProQuest)**

August 13, 2020; 1970-2020; English. Fields searched: title, abstract, keyword, subject.

(ti(cannabis OR marijuana OR hemp) OR ab(cannabis OR marijuana OR hemp) OR diskw(cannabis OR marijuana OR hemp) OR su(“cannabis” OR “cannabis edibles” OR “hemp” OR “marijuana” OR “medical marijuana”)) **AND** (ti(commercializ* OR “social club*” OR collective OR cooperative OR cultivat* OR decrim* OR dispensar* OR grow* OR industr* OR law* OR legal* OR legislat* OR liberaliz* OR medical* OR polic* OR program* OR recreation* OR regulat* OR retail*) OR ab(commercializ* OR “social club*” OR collective OR cooperative OR cultivat* OR decrim* OR dispensar* OR grow* OR industr* OR law* OR legal* OR legislat* OR liberaliz* OR medical* OR polic* OR program* OR recreation* OR regulat* OR retail*) OR diskw(commercializ* OR “social club*” OR collective OR cooperative OR cultivat* OR decrim* OR dispensar* OR grow* OR industr* OR law* OR legal* OR legislat* OR liberaliz* OR medical* OR polic* OR program* OR recreation* OR regulat* OR retail*) OR su(“commercialization” OR “drug control” OR “drug laws & regulations” OR “drug legalization” OR “government policy” OR “government regulation” OR “government regulations” OR “law” & “legalization” OR “legislation” OR “legislation, drug” OR “legislation, medical” OR “liberalization” OR “marijuana dispensaries” OR “marijuana growing” OR “marijuana industry” OR “marijuana laws” OR “marijuana legalization” OR “medical marijuana -- law & legislation” OR “medicalization” OR “public policy” OR “public policy (law)” OR “regulations” OR “retail stores” OR “state laws” OR “state regulation”)) **AND** (ti(analysis OR “before-and-after” OR causal OR counterfactual OR “difference-in-difference*” OR experiment* OR “fixed effect*” OR “instrumental variable*” OR longitudinal OR matching OR meta-analy* OR model* OR “panel data” OR “propensity score” OR “propensity match*” OR quasi-experiment* OR regress* OR “regression discontinuity” OR “repeated measures” OR RCT OR “synthetic control” OR “systematic review” OR “time-series”) OR ab(analysis OR “before-and-after” OR causal OR counterfactual OR “difference-in-difference*” OR experiment* OR “fixed effect*” OR “instrumental variable*” OR longitudinal OR matching OR meta-analy* OR model* OR “panel data” OR “propensity score” OR “propensity match*” OR quasi-experiment* OR regress* OR “regression discontinuity” OR “repeated measures” OR RCT OR “synthetic control” OR “systematic review” OR “time-series”) OR diskw(analysis OR “before-and-after” OR causal OR counterfactual OR “difference-in-difference*” OR experiment* OR “fixed effect*” OR “instrumental variable*” OR longitudinal OR matching OR meta-analy* OR model* OR “panel data” OR “propensity score” OR “propensity match*” OR quasi-experiment* OR regress* OR “regression discontinuity” OR “repeated measures” OR RCT OR “synthetic control” OR “systematic review” OR “time-series”) OR su(“causal modeling” OR “causal models” OR “controlled before-after studies” OR “controlled before and after studies” OR “data analysis” OR “data analysis, statistical” OR “econometric models” OR “fixed effects model” OR “granger causality test” OR “instrumental variables (statistics)” OR “instrumental variable estimation” OR “interrupted time series analysis” OR “longitudinal method” OR “longitudinal studies” OR “meta-analysis” OR “panel analysis” OR “panel data” OR “policy analysis” OR “population-based case control” OR “pretests posttests” OR “pretest-posttest design” OR “propensity score” OR “propensity score matching” OR “statistical analysis” OR “statistical matching” OR “quantitative research” OR “quasi-experimental studies” OR “regression” OR “regression analysis” OR “regression discontinuity design” OR “repeated measures” OR “repeated measures design” OR “time series” OR “time series analysis”))

**(10) EconLit (EBSCO)**

August 13, 2020; 1992-2020; English. Fields searched: title, abstract, subject.

(TI (cannabis OR marijuana OR hemp) OR AB (cannabis OR marijuana OR hemp) OR SU (“cannabis” OR “cannabis edibles” OR “hemp” OR “marijuana” OR “medical marijuana”)) **AND** (TI (commercializ* OR “social club*” OR collective OR cooperative OR cultivat* OR decrim* OR dispensar* OR grow* OR industr* OR law* OR legal* OR legislat* OR liberaliz* OR medical* OR polic* OR program* OR recreation* OR regulat* OR retail*) OR AB (commercializ* OR “social club*” OR collective OR cooperative OR cultivat* OR decrim* OR dispensar* OR grow* OR industr* OR law* OR legal* OR legislat* OR liberaliz* OR medical* OR polic* OR program* OR recreation* OR regulat* OR retail*) OR SU (“commercialization” OR “drug control” OR “drug laws & regulations” OR “drug legalization” OR “government policy” OR “government regulation” OR “government regulations” OR “law” & “legalization” OR “legislation” OR “legislation, drug” OR “legislation, medical” OR “liberalization” OR “marijuana dispensaries” OR “marijuana growing” OR “marijuana industry” OR “marijuana laws” OR “marijuana legalization” OR “medical marijuana -- law & legislation” OR “medicalization” OR “public policy” OR “public policy (law)” OR “regulations” OR “retail stores” OR “state laws” OR “state regulation”)) **AND** (TI (analysis OR “before-and-after” OR causal OR counterfactual OR “difference-in-difference*” OR experiment* OR “fixed effect*” OR “instrumental variable*” OR longitudinal OR matching OR meta-analy* OR model* OR “panel data” OR “propensity score” OR “propensity match*” OR quasi-experiment* OR regress* OR “regression discontinuity” OR “repeated measures” OR RCT OR “synthetic control” OR “systematic review” OR “time-series”) OR AB (analysis OR “before-and-after” OR causal OR counterfactual OR “difference-in-difference*” OR experiment* OR “fixed effect*” OR “instrumental variable*” OR longitudinal OR matching OR meta-analy* OR model* OR “panel data” OR “propensity score” OR “propensity match*” OR quasi-experiment* OR regress* OR “regression discontinuity” OR “repeated measures” OR RCT OR “synthetic control” OR “systematic review” OR “time-series”) OR SU (“causal modeling” OR “causal models” OR “controlled before-after studies” OR “controlled before and after studies” OR “data analysis” OR “data analysis, statistical” OR “econometric models” OR “fixed effects model” OR “granger causality test” OR “instrumental variables (statistics)” OR “instrumental variable estimation” OR “interrupted time series analysis” OR “longitudinal method” OR “longitudinal studies” OR “meta-analysis” OR “panel analysis” OR “panel data” OR “policy analysis” OR “population-based case control” OR “pretests posttests” OR “pretest-posttest design” OR “propensity score” OR “propensity score matching” OR “statistical analysis” OR “statistical matching” OR “quantitative research” OR “quasi-experimental studies” OR “regression” OR “regression analysis” OR “regression discontinuity design” OR “repeated measures” OR “repeated measures design” OR “time series” OR “time series analysis”))

**(11) Embase (Elsevier)**

August 13, 2020; 1970-2020; English. Fields searched: title, abstract, keyword, subject.

((cannabis:ti,ab,kw OR marijuana:ti,ab,kw OR hemp:ti,ab,kw) AND (commercializ*:ti,ab,kw OR 'social club*':ti,ab,kw OR collective:ti,ab,kw OR cooperative:ti,ab,kw OR cultivat*:ti,ab,kw OR decrim*:ti,ab,kw OR dispensar*:ti,ab,kw OR grow*:ti,ab,kw OR industr*:ti,ab,kw OR law*:ti,ab,kw OR legal*:ti,ab,kw OR legislat*:ti,ab,kw OR liberaliz*:ti,ab,kw OR medical*:ti,ab,kw OR polic*:ti,ab,kw OR program*:ti,ab,kw OR recreation*:ti,ab,kw OR regulat*:ti,ab,kw OR retail*:ti,ab,kw) AND (analysis:ti,ab,kw OR 'before-and-after':ti,ab,kw OR causal:ti,ab,kw OR counterfactual:ti,ab,kw OR 'difference-in-difference*':ti,ab,kw OR experiment*:ti,ab,kw OR 'fixed effect*':ti,ab,kw OR 'instrumental variable*':ti,ab,kw OR longitudinal:ti,ab,kw OR matching:ti,ab,kw OR 'meta analy*':ti,ab,kw OR model*:ti,ab,kw OR 'panel data':ti,ab,kw OR 'propensity score':ti,ab,kw OR 'propensity match*':ti,ab,kw OR 'quasi experiment*':ti,ab,kw OR regress*:ti,ab,kw OR 'regression discontinuity':ti,ab,kw OR 'repeated measures':ti,ab,kw OR rct:ti,ab,kw OR 'synthetic control':ti,ab,kw OR 'systematic review':ti,ab,kw OR 'time-series':ti,ab,kw)) OR (('cannabis'/de OR 'medical cannabis'/de) AND 'drug legislation'/de)

**(12) ERIC (EBSCO)**

August 13, 2020; 1970-2020; English. Fields searched: title, abstract, keyword, subject.

(TI (cannabis OR marijuana OR hemp) OR AB (cannabis OR marijuana OR hemp) OR KW (cannabis OR marijuana OR hemp) OR SU (“cannabis” OR “cannabis edibles” OR “hemp” OR “marijuana” OR “medical marijuana”)) **AND** (TI (commercializ* OR “social club*” OR collective OR cooperative OR cultivat* OR decrim* OR dispensar* OR grow* OR industr* OR law* OR legal* OR legislat* OR liberaliz* OR medical* OR polic* OR program* OR recreation* OR regulat* OR retail*) OR AB (commercializ* OR “social club*” OR collective OR cooperative OR cultivat* OR decrim* OR dispensar* OR grow* OR industr* OR law* OR legal* OR legislat* OR liberaliz* OR medical* OR polic* OR program* OR recreation* OR regulat* OR retail*) OR KW (commercializ* OR “social club*” OR collective OR cooperative OR cultivat* OR decrim* OR dispensar* OR grow* OR industr* OR law* OR legal* OR legislat* OR liberaliz* OR medical* OR polic* OR program* OR recreation* OR regulat* OR retail*) OR SU (“commercialization” OR “drug control” OR “drug laws & regulations” OR “drug legalization” OR “government policy” OR “government regulation” OR “government regulations” OR “law” & “legalization” OR “legislation” OR “legislation, drug” OR “legislation, medical” OR “liberalization” OR “marijuana dispensaries” OR “marijuana growing” OR “marijuana industry” OR “marijuana laws” OR “marijuana legalization” OR “medical marijuana -- law & legislation” OR “medicalization” OR “public policy” OR “public policy (law)” OR “regulations” OR “retail stores” OR “state laws” OR “state regulation”)) **AND** (TI (analysis OR “before-and-after” OR causal OR counterfactual OR “difference-in-difference*” OR experiment* OR “fixed effect*” OR “instrumental variable*” OR longitudinal OR matching OR meta-analy* OR model* OR “panel data” OR “propensity score” OR “propensity match*” OR quasi-experiment* OR regress* OR “regression discontinuity” OR “repeated measures” OR RCT OR “synthetic control” OR “systematic review” OR “time-series”) OR AB (analysis OR “before-and-after” OR causal OR counterfactual OR “difference-in-difference*” OR experiment* OR “fixed effect*” OR “instrumental variable*” OR longitudinal OR matching OR meta-analy* OR model* OR “panel data” OR “propensity score” OR “propensity match*” OR quasi-experiment* OR regress* OR “regression discontinuity” OR “repeated measures” OR RCT OR “synthetic control” OR “systematic review” OR “time-series”) OR KW (analysis OR “before-and-after” OR causal OR counterfactual OR “difference-in-difference*” OR experiment* OR “fixed effect*” OR “instrumental variable*” OR longitudinal OR matching OR meta-analy* OR model* OR “panel data” OR “propensity score” OR “propensity match*” OR quasi-experiment* OR regress* OR “regression discontinuity” OR “repeated measures” OR RCT OR “synthetic control” OR “systematic review” OR “time-series”) OR SU (“causal modeling” OR “causal models” OR “controlled before-after studies” OR “controlled before and after studies” OR “data analysis” OR “data analysis, statistical” OR “econometric models” OR “fixed effects model” OR “granger causality test” OR “instrumental variables (statistics)” OR “instrumental variable estimation” OR “interrupted time series analysis” OR “longitudinal method” OR “longitudinal studies” OR “meta-analysis” OR “panel analysis” OR “panel data” OR “policy analysis” OR “population-based case control” OR “pretests posttests” OR “pretest-posttest design” OR “propensity score” OR “propensity score matching” OR “statistical analysis” OR “statistical matching” OR “quantitative research” OR “quasi-experimental studies” OR “regression” OR “regression analysis” OR “regression discontinuity design” OR “repeated measures” OR “repeated measures design” OR “time series” OR “time series analysis”))

**(13) Healthcare Administration Database (ProQuest)**

August 13, 2020; 1980-2020; Publication types: Excludes wire feeds, newspapers, trade journals, magazines; English. Fields searched: title, abstract, subject.

(ti(cannabis OR marijuana OR hemp) OR ab(cannabis OR marijuana OR hemp) OR su(“cannabis” OR “cannabis edibles” OR “hemp” OR “marijuana” OR “medical marijuana”)) **AND** (ti(commercializ* OR “social club*” OR collective OR cooperative OR cultivat* OR decrim* OR dispensar* OR grow* OR industr* OR law* OR legal* OR legislat* OR liberaliz* OR medical* OR polic* OR program* OR recreation* OR regulat* OR retail*) OR ab(commercializ* OR “social club*” OR collective OR cooperative OR cultivat* OR decrim* OR dispensar* OR grow* OR industr* OR law* OR legal* OR legislat* OR liberaliz* OR medical* OR polic* OR program* OR recreation* OR regulat* OR retail*) OR su(“commercialization” OR “drug control” OR “drug laws & regulations” OR “drug legalization” OR “government policy” OR “government regulation” OR “government regulations” OR “law” & “legalization” OR “legislation” OR “legislation, drug” OR “legislation, medical” OR “liberalization” OR “marijuana dispensaries” OR “marijuana growing” OR “marijuana industry” OR “marijuana laws” OR “marijuana legalization” OR “medical marijuana -- law & legislation” OR “medicalization” OR “public policy” OR “public policy (law)” OR “regulations” OR “retail stores” OR “state laws” OR “state regulation”)) **AND** (ti(analysis OR “before-and-after” OR causal OR counterfactual OR “difference-in-difference*” OR experiment* OR “fixed effect*” OR “instrumental variable*” OR longitudinal OR matching OR meta-analy* OR model* OR “panel data” OR “propensity score” OR “propensity match*” OR quasi-experiment* OR regress* OR “regression discontinuity” OR “repeated measures” OR RCT OR “synthetic control” OR “systematic review” OR “time-series”) OR ab(analysis OR “before-and-after” OR causal OR counterfactual OR “difference-in-difference*” OR experiment* OR “fixed effect*” OR “instrumental variable*” OR longitudinal OR matching OR meta-analy* OR model* OR “panel data” OR “propensity score” OR “propensity match*” OR quasi-experiment* OR regress* OR “regression discontinuity” OR “repeated measures” OR RCT OR “synthetic control” OR “systematic review” OR “time-series”) OR su(“causal modeling” OR “causal models” OR “controlled before-after studies” OR “controlled before and after studies” OR “data analysis” OR “data analysis, statistical” OR “econometric models” OR “fixed effects model” OR “granger causality test” OR “instrumental variables (statistics)” OR “instrumental variable estimation” OR “interrupted time series analysis” OR “longitudinal method” OR “longitudinal studies” OR “meta-analysis” OR “panel analysis” OR “panel data” OR “policy analysis” OR “population-based case control” OR “pretests posttests” OR “pretest-posttest design” OR “propensity score” OR “propensity score matching” OR “statistical analysis” OR “statistical matching” OR “quantitative research” OR “quasi-experimental studies” OR “regression” OR “regression analysis” OR “regression discontinuity design” OR “repeated measures” OR “repeated measures design” OR “time series” OR “time series analysis”))

**(14) Health Source: Nursing/Academic Edition (EBSCO)**

August 13, 2020; 1977-2020; Publication types: Excludes magazines, trade publications; English. Fields searched: title, abstract, keyword subject.

(TI (cannabis OR marijuana OR hemp) OR AB (cannabis OR marijuana OR hemp) OR KW (cannabis OR marijuana OR hemp) OR SU (“cannabis” OR “cannabis edibles” OR “hemp” OR “marijuana” OR “medical marijuana”)) **AND** (TI (commercializ* OR “social club*” OR collective OR cooperative OR cultivat* OR decrim* OR dispensar* OR grow* OR industr* OR law* OR legal* OR legislat* OR liberaliz* OR medical* OR polic* OR program* OR recreation* OR regulat* OR retail*) OR AB (commercializ* OR “social club*” OR collective OR cooperative OR cultivat* OR decrim* OR dispensar* OR grow* OR industr* OR law* OR legal* OR legislat* OR liberaliz* OR medical* OR polic* OR program* OR recreation* OR regulat* OR retail*) OR KW (commercializ* OR “social club*” OR collective OR cooperative OR cultivat* OR decrim* OR dispensar* OR grow* OR industr* OR law* OR legal* OR legislat* OR liberaliz* OR medical* OR polic* OR program* OR recreation* OR regulat* OR retail*) OR SU (“commercialization” OR “drug control” OR “drug laws & regulations” OR “drug legalization” OR “government policy” OR “government regulation” OR “government regulations” OR “law” & “legalization” OR “legislation” OR “legislation, drug” OR “legislation, medical” OR “liberalization” OR “marijuana dispensaries” OR “marijuana growing” OR “marijuana industry” OR “marijuana laws” OR “marijuana legalization” OR “medical marijuana -- law & legislation” OR “medicalization” OR “public policy” OR “public policy (law)” OR “regulations” OR “retail stores” OR “state laws” OR “state regulation”)) **AND** (TI (analysis OR “before-and-after” OR causal OR counterfactual OR “difference-in-difference*” OR experiment* OR “fixed effect*” OR “instrumental variable*” OR longitudinal OR matching OR meta-analy* OR model* OR “panel data” OR “propensity score” OR “propensity match*” OR quasi-experiment* OR regress* OR “regression discontinuity” OR “repeated measures” OR RCT OR “synthetic control” OR “systematic review” OR “time-series”) OR AB (analysis OR “before-and-after” OR causal OR counterfactual OR “difference-in-difference*” OR experiment* OR “fixed effect*” OR “instrumental variable*” OR longitudinal OR matching OR meta-analy* OR model* OR “panel data” OR “propensity score” OR “propensity match*” OR quasi-experiment* OR regress* OR “regression discontinuity” OR “repeated measures” OR RCT OR “synthetic control” OR “systematic review” OR “time-series”) OR KW (analysis OR “before-and-after” OR causal OR counterfactual OR “difference-in-difference*” OR experiment* OR “fixed effect*” OR “instrumental variable*” OR longitudinal OR matching OR meta-analy* OR model* OR “panel data” OR “propensity score” OR “propensity match*” OR quasi-experiment* OR regress* OR “regression discontinuity” OR “repeated measures” OR RCT OR “synthetic control” OR “systematic review” OR “time-series”) OR SU (“causal modeling” OR “causal models” OR “controlled before-after studies” OR “controlled before and after studies” OR “data analysis” OR “data analysis, statistical” OR “econometric models” OR “fixed effects model” OR “granger causality test” OR “instrumental variables (statistics)” OR “instrumental variable estimation” OR “interrupted time series analysis” OR “longitudinal method” OR “longitudinal studies” OR “meta-analysis” OR “panel analysis” OR “panel data” OR “policy analysis” OR “population-based case control” OR “pretests posttests” OR “pretest-posttest design” OR “propensity score” OR “propensity score matching” OR “statistical analysis” OR “statistical matching” OR “quantitative research” OR “quasi-experimental studies” OR “regression” OR “regression analysis” OR “regression discontinuity design” OR “repeated measures” OR “repeated measures design” OR “time series” OR “time series analysis”))

**(15) International Bibliography of the Social Sciences (ProQuest)**

August 14, 2020; 1980-2020; Publication types: Exclude magazines, newspapers; English. Fields searched: title, abstract, subject.

(ti(cannabis OR marijuana OR hemp) OR ab(cannabis OR marijuana OR hemp) OR su(“cannabis” OR “cannabis edibles” OR “hemp” OR “marijuana” OR “medical marijuana”)) **AND** (ti(commercializ* OR “social club*” OR collective OR cooperative OR cultivat* OR decrim* OR dispensar* OR grow* OR industr* OR law* OR legal* OR legislat* OR liberaliz* OR medical* OR polic* OR program* OR recreation* OR regulat* OR retail*) OR ab(commercializ* OR “social club*” OR collective OR cooperative OR cultivat* OR decrim* OR dispensar* OR grow* OR industr* OR law* OR legal* OR legislat* OR liberaliz* OR medical* OR polic* OR program* OR recreation* OR regulat* OR retail*) OR su(“commercialization” OR “drug control” OR “drug laws & regulations” OR “drug legalization” OR “government policy” OR “government regulation” OR “government regulations” OR “law” & “legalization” OR “legislation” OR “legislation, drug” OR “legislation, medical” OR “liberalization” OR “marijuana dispensaries” OR “marijuana growing” OR “marijuana industry” OR “marijuana laws” OR “marijuana legalization” OR “medical marijuana -- law & legislation” OR “medicalization” OR “public policy” OR “public policy (law)” OR “regulations” OR “retail stores” OR “state laws” OR “state regulation”)) **AND** (ti(analysis OR “before-and-after” OR causal OR counterfactual OR “difference-in-difference*” OR experiment* OR “fixed effect*” OR “instrumental variable*” OR longitudinal OR matching OR meta-analy* OR model* OR “panel data” OR “propensity score” OR “propensity match*” OR quasi-experiment* OR regress* OR “regression discontinuity” OR “repeated measures” OR RCT OR “synthetic control” OR “systematic review” OR “time-series”) OR ab(analysis OR “before-and-after” OR causal OR counterfactual OR “difference-in-difference*” OR experiment* OR “fixed effect*” OR “instrumental variable*” OR longitudinal OR matching OR meta-analy* OR model* OR “panel data” OR “propensity score” OR “propensity match*” OR quasi-experiment* OR regress* OR “regression discontinuity” OR “repeated measures” OR RCT OR “synthetic control” OR “systematic review” OR “time-series”) OR su(“causal modeling” OR “causal models” OR “controlled before-after studies” OR “controlled before and after studies” OR “data analysis” OR “data analysis, statistical” OR “econometric models” OR “fixed effects model” OR “granger causality test” OR “instrumental variables (statistics)” OR “instrumental variable estimation” OR “interrupted time series analysis” OR “longitudinal method” OR “longitudinal studies” OR “meta-analysis” OR “panel analysis” OR “panel data” OR “policy analysis” OR “population-based case control” OR “pretests posttests” OR “pretest-posttest design” OR “propensity score” OR “propensity score matching” OR “statistical analysis” OR “statistical matching” OR “quantitative research” OR “quasi-experimental studies” OR “regression” OR “regression analysis” OR “regression discontinuity design” OR “repeated measures” OR “repeated measures design” OR “time series” OR “time series analysis”))

**(16) Legal Source (EBSCO)**

August 14, 2020; 1970-2020; Publication types: Excludes magazines, book reviews; English. Fields searched: title, abstract, keyword subject.

(TI (cannabis OR marijuana OR hemp) OR AB (cannabis OR marijuana OR hemp) OR KW (cannabis OR marijuana OR hemp) OR SU (“cannabis” OR “cannabis edibles” OR “hemp” OR “marijuana” OR “medical marijuana”)) **AND** (TI (commercializ* OR “social club*” OR collective OR cooperative OR cultivat* OR decrim* OR dispensar* OR grow* OR industr* OR law* OR legal* OR legislat* OR liberaliz* OR medical* OR polic* OR program* OR recreation* OR regulat* OR retail*) OR AB (commercializ* OR “social club*” OR collective OR cooperative OR cultivat* OR decrim* OR dispensar* OR grow* OR industr* OR law* OR legal* OR legislat* OR liberaliz* OR medical* OR polic* OR program* OR recreation* OR regulat* OR retail*) OR KW (commercializ* OR “social club*” OR collective OR cooperative OR cultivat* OR decrim* OR dispensar* OR grow* OR industr* OR law* OR legal* OR legislat* OR liberaliz* OR medical* OR polic* OR program* OR recreation* OR regulat* OR retail*) OR SU (“commercialization” OR “drug control” OR “drug laws & regulations” OR “drug legalization” OR “government policy” OR “government regulation” OR “government regulations” OR “law” & “legalization” OR “legislation” OR “legislation, drug” OR “legislation, medical” OR “liberalization” OR “marijuana dispensaries” OR “marijuana growing” OR “marijuana industry” OR “marijuana laws” OR “marijuana legalization” OR “medical marijuana -- law & legislation” OR “medicalization” OR “public policy” OR “public policy (law)” OR “regulations” OR “retail stores” OR “state laws” OR “state regulation”)) **AND** (TI (analysis OR “before-and-after” OR causal OR counterfactual OR “difference-in-difference*” OR experiment* OR “fixed effect*” OR “instrumental variable*” OR longitudinal OR matching OR meta-analy* OR model* OR “panel data” OR “propensity score” OR “propensity match*” OR quasi-experiment* OR regress* OR “regression discontinuity” OR “repeated measures” OR RCT OR “synthetic control” OR “systematic review” OR “time-series”) OR AB (analysis OR “before-and-after” OR causal OR counterfactual OR “difference-in-difference*” OR experiment* OR “fixed effect*” OR “instrumental variable*” OR longitudinal OR matching OR meta-analy* OR model* OR “panel data” OR “propensity score” OR “propensity match*” OR quasi-experiment* OR regress* OR “regression discontinuity” OR “repeated measures” OR RCT OR “synthetic control” OR “systematic review” OR “time-series”) OR KW (analysis OR “before-and-after” OR causal OR counterfactual OR “difference-in-difference*” OR experiment* OR “fixed effect*” OR “instrumental variable*” OR longitudinal OR matching OR meta-analy* OR model* OR “panel data” OR “propensity score” OR “propensity match*” OR quasi-experiment* OR regress* OR “regression discontinuity” OR “repeated measures” OR RCT OR “synthetic control” OR “systematic review” OR “time-series”) OR SU (“causal modeling” OR “causal models” OR “controlled before-after studies” OR “controlled before and after studies” OR “data analysis” OR “data analysis, statistical” OR “econometric models” OR “fixed effects model” OR “granger causality test” OR “instrumental variables (statistics)” OR “instrumental variable estimation” OR “interrupted time series analysis” OR “longitudinal method” OR “longitudinal studies” OR “meta-analysis” OR “panel analysis” OR “panel data” OR “policy analysis” OR “population-based case control” OR “pretests posttests” OR “pretest-posttest design” OR “propensity score” OR “propensity score matching” OR “statistical analysis” OR “statistical matching” OR “quantitative research” OR “quasi-experimental studies” OR “regression” OR “regression analysis” OR “regression discontinuity design” OR “repeated measures” OR “repeated measures design” OR “time series” OR “time series analysis”))

**(17) MEDLINE (EBSCO)**

August 14, 2020; 1971-2020; Publication types: Excludes magazines, guidelines; English. Fields searched: title, abstract, subject.

(TI (cannabis OR marijuana OR hemp) OR AB (cannabis OR marijuana OR hemp) OR SU (“cannabis” OR “cannabis edibles” OR “hemp” OR “marijuana” OR “medical marijuana”)) **AND** (TI (commercializ* OR “social club*” OR collective OR cooperative OR cultivat* OR decrim* OR dispensar* OR grow* OR industr* OR law* OR legal* OR legislat* OR liberaliz* OR medical* OR polic* OR program* OR recreation* OR regulat* OR retail*) OR AB (commercializ* OR “social club*” OR collective OR cooperative OR cultivat* OR decrim* OR dispensar* OR grow* OR industr* OR law* OR legal* OR legislat* OR liberaliz* OR medical* OR polic* OR program* OR recreation* OR regulat* OR retail*) OR SU (“commercialization” OR “drug control” OR “drug laws & regulations” OR “drug legalization” OR “government policy” OR “government regulation” OR “government regulations” OR “law” & “legalization” OR “legislation” OR “legislation, drug” OR “legislation, medical” OR “liberalization” OR “marijuana dispensaries” OR “marijuana growing” OR “marijuana industry” OR “marijuana laws” OR “marijuana legalization” OR “medical marijuana -- law & legislation” OR “medicalization” OR “public policy” OR “public policy (law)” OR “regulations” OR “retail stores” OR “state laws” OR “state regulation”)) **AND** (TI (analysis OR “before-and-after” OR causal OR counterfactual OR “difference-in-difference*” OR experiment* OR “fixed effect*” OR “instrumental variable*” OR longitudinal OR matching OR meta-analy* OR model* OR “panel data” OR “propensity score” OR “propensity match*” OR quasi-experiment* OR regress* OR “regression discontinuity” OR “repeated measures” OR RCT OR “synthetic control” OR “systematic review” OR “time-series”) OR AB (analysis OR “before-and-after” OR causal OR counterfactual OR “difference-in-difference*” OR experiment* OR “fixed effect*” OR “instrumental variable*” OR longitudinal OR matching OR meta-analy* OR model* OR “panel data” OR “propensity score” OR “propensity match*” OR quasi-experiment* OR regress* OR “regression discontinuity” OR “repeated measures” OR RCT OR “synthetic control” OR “systematic review” OR “time-series”) OR SU (“causal modeling” OR “causal models” OR “controlled before-after studies” OR “controlled before and after studies” OR “data analysis” OR “data analysis, statistical” OR “econometric models” OR “fixed effects model” OR “granger causality test” OR “instrumental variables (statistics)” OR “instrumental variable estimation” OR “interrupted time series analysis” OR “longitudinal method” OR “longitudinal studies” OR “meta-analysis” OR “panel analysis” OR “panel data” OR “policy analysis” OR “population-based case control” OR “pretests posttests” OR “pretest-posttest design” OR “propensity score” OR “propensity score matching” OR “statistical analysis” OR “statistical matching” OR “quantitative research” OR “quasi-experimental studies” OR “regression” OR “regression analysis” OR “regression discontinuity design” OR “repeated measures” OR “repeated measures design” OR “time series” OR “time series analysis”))

**(18) National Criminal Justice Reference Service Abstracts Database (ProQuest)**

August 14, 2020; 1970-2019; Publication types: Excludes magazines; English. Fields searched: title, abstract, subject.

(ti(cannabis OR marijuana OR hemp) OR ab(cannabis OR marijuana OR hemp) OR su(“cannabis” OR “cannabis edibles” OR “hemp” OR “marijuana” OR “medical marijuana”)) **AND** (ti(commercializ* OR “social club*” OR collective OR cooperative OR cultivat* OR decrim* OR dispensar* OR grow* OR industr* OR law* OR legal* OR legislat* OR liberaliz* OR medical* OR polic* OR program* OR recreation* OR regulat* OR retail*) OR ab(commercializ* OR “social club*” OR collective OR cooperative OR cultivat* OR decrim* OR dispensar* OR grow* OR industr* OR law* OR legal* OR legislat* OR liberaliz* OR medical* OR polic* OR program* OR recreation* OR regulat* OR retail*) OR su(“commercialization” OR “drug control” OR “drug laws & regulations” OR “drug legalization” OR “government policy” OR “government regulation” OR “government regulations” OR “law” & “legalization” OR “legislation” OR “legislation, drug” OR “legislation, medical” OR “liberalization” OR “marijuana dispensaries” OR “marijuana growing” OR “marijuana industry” OR “marijuana laws” OR “marijuana legalization” OR “medical marijuana -- law & legislation” OR “medicalization” OR “public policy” OR “public policy (law)” OR “regulations” OR “retail stores” OR “state laws” OR “state regulation”)) **AND** (ti(analysis OR “before-and-after” OR causal OR counterfactual OR “difference-in-difference*” OR experiment* OR “fixed effect*” OR “instrumental variable*” OR longitudinal OR matching OR meta-analy* OR model* OR “panel data” OR “propensity score” OR “propensity match*” OR quasi-experiment* OR regress* OR “regression discontinuity” OR “repeated measures” OR RCT OR “synthetic control” OR “systematic review” OR “time-series”) OR ab(analysis OR “before-and-after” OR causal OR counterfactual OR “difference-in-difference*” OR experiment* OR “fixed effect*” OR “instrumental variable*” OR longitudinal OR matching OR meta-analy* OR model* OR “panel data” OR “propensity score” OR “propensity match*” OR quasi-experiment* OR regress* OR “regression discontinuity” OR “repeated measures” OR RCT OR “synthetic control” OR “systematic review” OR “time-series”) OR su(“causal modeling” OR “causal models” OR “controlled before-after studies” OR “controlled before and after studies” OR “data analysis” OR “data analysis, statistical” OR “econometric models” OR “fixed effects model” OR “granger causality test” OR “instrumental variables (statistics)” OR “instrumental variable estimation” OR “interrupted time series analysis” OR “longitudinal method” OR “longitudinal studies” OR “meta-analysis” OR “panel analysis” OR “panel data” OR “policy analysis” OR “population-based case control” OR “pretests posttests” OR “pretest-posttest design” OR “propensity score” OR “propensity score matching” OR “statistical analysis” OR “statistical matching” OR “quantitative research” OR “quasi-experimental studies” OR “regression” OR “regression analysis” OR “regression discontinuity design” OR “repeated measures” OR “repeated measures design” OR “time series” OR “time series analysis”))

**(19) PAIS Index (ProQuest)**

August 14, 2020; 1976-2020; Publication types: Excludes magazines, trade journals; English. Fields searched: title, abstract, keyword, subject.

(ti(cannabis OR marijuana OR hemp) OR ab(cannabis OR marijuana OR hemp) OR if(cannabis OR marijuana OR hemp) OR su(“cannabis” OR “cannabis edibles” OR “hemp” OR “marijuana” OR “medical marijuana”)) **AND** (ti(commercializ* OR “social club*” OR collective OR cooperative OR cultivat* OR decrim* OR dispensar* OR grow* OR industr* OR law* OR legal* OR legislat* OR liberaliz* OR medical* OR polic* OR program* OR recreation* OR regulat* OR retail*) OR ab(commercializ* OR “social club*” OR collective OR cooperative OR cultivat* OR decrim* OR dispensar* OR grow* OR industr* OR law* OR legal* OR legislat* OR liberaliz* OR medical* OR polic* OR program* OR recreation* OR regulat* OR retail*) OR if(commercializ* OR “social club*” OR collective OR cooperative OR cultivat* OR decrim* OR dispensar* OR grow* OR industr* OR law* OR legal* OR legislat* OR liberaliz* OR medical* OR polic* OR program* OR recreation* OR regulat* OR retail*) OR su(“commercialization” OR “drug control” OR “drug laws & regulations” OR “drug legalization” OR “government policy” OR “government regulation” OR “government regulations” OR “law” & “legalization” OR “legislation” OR “legislation, drug” OR “legislation, medical” OR “liberalization” OR “marijuana dispensaries” OR “marijuana growing” OR “marijuana industry” OR “marijuana laws” OR “marijuana legalization” OR “medical marijuana -- law & legislation” OR “medicalization” OR “public policy” OR “public policy (law)” OR “regulations” OR “retail stores” OR “state laws” OR “state regulation”)) **AND** (ti(analysis OR “before-and-after” OR causal OR counterfactual OR “difference-in-difference*” OR experiment* OR “fixed effect*” OR “instrumental variable*” OR longitudinal OR matching OR meta-analy* OR model* OR “panel data” OR “propensity score” OR “propensity match*” OR quasi-experiment* OR regress* OR “regression discontinuity” OR “repeated measures” OR RCT OR “synthetic control” OR “systematic review” OR “time-series”) OR ab(analysis OR “before-and-after” OR causal OR counterfactual OR “difference-in-difference*” OR experiment* OR “fixed effect*” OR “instrumental variable*” OR longitudinal OR matching OR meta-analy* OR model* OR “panel data” OR “propensity score” OR “propensity match*” OR quasi-experiment* OR regress* OR “regression discontinuity” OR “repeated measures” OR RCT OR “synthetic control” OR “systematic review” OR “time-series”) OR if(analysis OR “before-and-after” OR causal OR counterfactual OR “difference-in-difference*” OR experiment* OR “fixed effect*” OR “instrumental variable*” OR longitudinal OR matching OR meta-analy* OR model* OR “panel data” OR “propensity score” OR “propensity match*” OR quasi-experiment* OR regress* OR “regression discontinuity” OR “repeated measures” OR RCT OR “synthetic control” OR “systematic review” OR “time-series”) OR su(“causal modeling” OR “causal models” OR “controlled before-after studies” OR “controlled before and after studies” OR “data analysis” OR “data analysis, statistical” OR “econometric models” OR “fixed effects model” OR “granger causality test” OR “instrumental variables (statistics)” OR “instrumental variable estimation” OR “interrupted time series analysis” OR “longitudinal method” OR “longitudinal studies” OR “meta-analysis” OR “panel analysis” OR “panel data” OR “policy analysis” OR “population-based case control” OR “pretests posttests” OR “pretest-posttest design” OR “propensity score” OR “propensity score matching” OR “statistical analysis” OR “statistical matching” OR “quantitative research” OR “quasi-experimental studies” OR “regression” OR “regression analysis” OR “regression discontinuity design” OR “repeated measures” OR “repeated measures design” OR “time series” OR “time series analysis”))

**(20) PROSPERO (University of York)**

August 14, 2020; no filters, all fields.

(cannabis OR marijuana OR hemp)

**(21) Research Library (ProQuest)**

August 14, 2020; 1970-2020; Publication types: Excludes newspapers, magazines, trade journals, wire feeds, blogs/podcasts/websites; English. Fields searched: title, abstract, subject.

(ti(cannabis OR marijuana OR hemp) OR ab(cannabis OR marijuana OR hemp) OR su(“cannabis” OR “cannabis edibles” OR “hemp” OR “marijuana” OR “medical marijuana”)) **AND** (ti(commercializ* OR “social club*” OR collective OR cooperative OR cultivat* OR decrim* OR dispensar* OR grow* OR industr* OR law* OR legal* OR legislat* OR liberaliz* OR medical* OR polic* OR program* OR recreation* OR regulat* OR retail*) OR ab(commercializ* OR “social club*” OR collective OR cooperative OR cultivat* OR decrim* OR dispensar* OR grow* OR industr* OR law* OR legal* OR legislat* OR liberaliz* OR medical* OR polic* OR program* OR recreation* OR regulat* OR retail*) OR su(“commercialization” OR “drug control” OR “drug laws & regulations” OR “drug legalization” OR “government policy” OR “government regulation” OR “government regulations” OR “law” & “legalization” OR “legislation” OR “legislation, drug” OR “legislation, medical” OR “liberalization” OR “marijuana dispensaries” OR “marijuana growing” OR “marijuana industry” OR “marijuana laws” OR “marijuana legalization” OR “medical marijuana -- law & legislation” OR “medicalization” OR “public policy” OR “public policy (law)” OR “regulations” OR “retail stores” OR “state laws” OR “state regulation”)) **AND** (ti(analysis OR “before-and-after” OR causal OR counterfactual OR “difference-in-difference*” OR experiment* OR “fixed effect*” OR “instrumental variable*” OR longitudinal OR matching OR meta-analy* OR model* OR “panel data” OR “propensity score” OR “propensity match*” OR quasi-experiment* OR regress* OR “regression discontinuity” OR “repeated measures” OR RCT OR “synthetic control” OR “systematic review” OR “time-series”) OR ab(analysis OR “before-and-after” OR causal OR counterfactual OR “difference-in-difference*” OR experiment* OR “fixed effect*” OR “instrumental variable*” OR longitudinal OR matching OR meta-analy* OR model* OR “panel data” OR “propensity score” OR “propensity match*” OR quasi-experiment* OR regress* OR “regression discontinuity” OR “repeated measures” OR RCT OR “synthetic control” OR “systematic review” OR “time-series”) OR su(“causal modeling” OR “causal models” OR “controlled before-after studies” OR “controlled before and after studies” OR “data analysis” OR “data analysis, statistical” OR “econometric models” OR “fixed effects model” OR “granger causality test” OR “instrumental variables (statistics)” OR “instrumental variable estimation” OR “interrupted time series analysis” OR “longitudinal method” OR “longitudinal studies” OR “meta-analysis” OR “panel analysis” OR “panel data” OR “policy analysis” OR “population-based case control” OR “pretests posttests” OR “pretest-posttest design” OR “propensity score” OR “propensity score matching” OR “statistical analysis” OR “statistical matching” OR “quantitative research” OR “quasi-experimental studies” OR “regression” OR “regression analysis” OR “regression discontinuity design” OR “repeated measures” OR “repeated measures design” OR “time series” OR “time series analysis”))

**(22) ScienceDirect (Elsevier)**

August 14, 2020; 1970-2020; Fields searched: title, abstract, keywords.

ScienceDirect only allows up to eight Boolean operators and does not support wildcard truncation. The following search string was used.

(cannabis OR marijuana OR hemp) AND (law OR legalization OR medical OR recreational OR decriminalization)

**(23) Web of Science: Core Collection: Citation Indexes**

August 14, 2020; 1985-2020; English; Indexes: SCI-EXPANDED, SSCI, A&HCI, CPCI-S, CPCI-SSH, BKCI-S, BKCI-SSH, ESCI.

((TS=(cannabis OR marijuana OR hemp) AND TS=(commercializ* OR “cannabis social club*” OR cultivat* OR decrim* OR dispensar* OR grow* OR industr* OR law* OR legal* OR legislat* OR liberaliz* OR medical* OR polic* OR program* OR recreation* OR regulat* OR retail*) AND TS=(analysis OR “before-and-after” OR causal OR counterfactual OR “difference-in-difference*” OR experiment* OR “fixed effect*” OR “instrumental variable*” OR longitudinal OR matching OR meta-analy* OR model* OR “panel data” OR “propensity score” OR “propensity match*” OR quasi-experiment* OR regress* OR “regression discontinuity” OR “repeated measures” OR RCT OR “synthetic control” OR “systematic review” OR “time-series”)))

**(24) Don M. Gottfredson Library of Criminal Justice Gray Literature Database**

(<https://njlaw.rutgers.edu/cj/gray/index.php>)

August 14, 2020

Searched ‘marijuana’, ‘cannabis, and ‘hemp’ separately in each Title and Subject Keyword fields.

**(25) European Monitoring Centre for Drugs and Drug Addiction**

(<https://www.emcdda.europa.eu/publications>)

August 14, 2020

Searched ‘cannabis’, ‘marijuana’, and ‘hemp’ separately in general field.

**(26) International Society for the Study of Drug Policy Grey Literature Bibliography** (<https://www.issdp.org/bibliography-introduction/>)

August 14, 2020

Searched ‘cannabis’, ‘marijuana’, and ‘hemp’ separately in general field.

**(27) IZA – Institute of Labor Economics**

(<https://www.iza.org/publications>)

August 14, 2020

Searched ‘marijuana’, ‘cannabis, and ‘hemp’ separately in each Title, Abstract, and Keyword fields.

**(28) National Bureau of Economic Research Working Papers**

(<https://www.nber.org/papers.html>)

August 14, 2020; Research outputs includes working papers, books, and conferences and meetings.

Searched following string: (“medical marijuana” OR “recreational marijuana” OR “marijuana legalization” OR “marijuana decriminalization” OR “medical cannabis” OR “recreational cannabis” OR “cannabis legalization” OR “cannabis decriminalization”)

**(29) RAND Corporation**

(<https://www.rand.org/search/advanced-search.html>)

August 14, 2020; Documents

Search: (marijuana OR cannabis OR hemp) AND (medical OR recreational OR decriminalization OR legalization)

**(30) National Drug and Alcohol Research Centre**

(<https://ndarc.med.unsw.edu.au/resources>)

August 14, 2020

Searched ‘marijuana’, ‘cannabis, and ‘hemp’ separately in main search field.

**(31) Open Society Framework**

(<https://osf.io/preprints/>)

August 14, 2020

Search: (marijuana OR cannabis OR hemp) AND (medical OR recreational OR decriminalization OR legalization)

**(32) RePEc**

(<https://ideas.repec.org/>)

August 15, 2020

Search: (marijuana OR cannabis OR hemp) AND (medical OR recreational OR decriminalization OR legalization)

**(33) ScholarWorks**

(<https://scholarworks.gsu.edu/>)

August 15, 2020

Search: (marijuana OR cannabis OR hemp) AND (commercializ* OR “cannabis social club*” OR cultivat* OR decrim* OR dispensar* OR grow* OR industr* OR law* OR legal* OR legislat* OR liberaliz* OR medical* OR polic* OR program* OR recreation* OR regulat* OR retail*) AND (analysis OR “before-and-after” OR causal OR counterfactual OR “difference-in-difference*” OR experiment* OR “fixed effect*” OR “instrumental variable*” OR longitudinal OR matching OR meta-analy* OR model* OR “panel data” OR “propensity score” OR “propensity match*” OR quasi-experiment* OR regress* OR “regression discontinuity” OR “repeated measures” OR RCT OR “synthetic control” OR “systematic review” OR “time-series”) in Title, Abstract, and Subject.

**(34) Social Sciences Research Network**

(<https://papers.ssrn.com/sol3/DisplayAbstractSearch.cfm>)

August 15, 2020

Searched ‘cannabis’, ‘marijuana’, and ‘hemp’ separately in Title, Abstract, and Keywords.

## 2 EGM coding guide

**Study Characteristics**

1. Target Country

- Australia
- Canada
- Czech Republic
- Italy
- Mexico
- Netherlands
- United States
- Uruguay

2. Study Time Period

- Pre-1990
- 1990-1999
- 2000-2009
- 2010-2019
- Not reported

3. Publication Type

- Abstract
- Dissertation/thesis
- Journal article
- Protocol
- Working paper

4. Funding Source

- Not reported
- Unfunded
- Funded

**Policies/Provisions**

5. Major Type of Law

- Comprehensive medical cannabis law
- Limited medical CBD law
- Recreational cannabis law
- Decriminalization of cannabis cultivation
- Industrial hemp law

6. Specific Medical Cannabis Law Provisions

- General law
- Policy index
- Patient registry
- Patient qualifying conditions
- Patient minimum legal age
- Patient home cultivation
- Patient plant/possession limits
- Patient employment protections
- Physician/provider regulations
- Caregiver regulations
- Social clubs and collectives
- Cultivation and production regulations
- Retail sales outlets
- Retail sales delivery services
- Retail sales limits
- Product and marketing regulations

7. Specific Recreational Cannabis Law Provisions

- General law
- Policy index
- User registry
- User minimum legal age
- User home cultivation
- User plant/possession limits
- Social clubs and collectives
- Cultivation and production regulations
- Retail sales outlets
- Retail sales delivery services
- Retail sales limits
- Product and marketing regulations

8. Policy Data Source

- Alcohol Policy Information System (APIS) – Recreational Use of Cannabis
- LawAtlas
- Marijuana Policy Project (MPP)
- National Conference of State Legislators (NCSL)
- National Organization for the Reform of Marijuana Laws (NORML)
- Prescription Drug Abuse Policy System (PDAPS)
- ProCon.org
- Other (specify)

**Outcomes**

9. Markets and Environments – Drug Market Characteristics

- Availability of cannabis
- Availability of other drugs
- Cannabis sales/revenues
- Cannabis potency
- Cannabis prices
- Other drug prices
- Illicit market diversion
- Illegal cannabis grows
- Criminal network size

10. Markets and Environments – Law Enforcement Activities

- Police interventions
- Differential enforcement

11. Markets and Environments – Advertising and Marketing

- Cannabis advertising exposure and marketing
- Other drug advertising exposure and marketing

12. Attitudes and Behaviors – Attitudes and Perceptions

- Interest in cannabis
- Interest in other drugs
- Attitudes toward cannabis
- Attitudes toward other drugs
- Risk perceptions of cannabis
- Risk perceptions of other drugs

13. Attitudes and Behaviors – Substance Use

- Cannabis use
- Opioid use
- Sedative use
- Stimulant use
- Alcohol use
- Tobacco use
- Prescription drug use
- Illicit drug use (general)
- Modes of cannabis consumption

14. Attitudes and Behaviors – Other Behaviors and Practices

- Food consumption
- Sexual activity
- Safe sex practices
- Physician medical practices

15. Public Health – Mortality

- Opioid-related mortality
- Alcohol-related mortality
- Drug-related mortality (general)
- Mortality (all-cause)

16. Public Health – Poisoning and Overdose

- Opioid overdose
- Alcohol overdose
- Drug overdose (general)
- Cannabis exposures
- Other drug exposures
- Hospital admissions (general)

17. Public Health – Substance Use Disorders

- Cannabis use disorders
- Alcohol use disorders
- Opioid use disorders
- Substance use disorders (general)
- Drug treatment adherence

18. Public Health – Mental Health

- Mental health (general)
- Mood disorders
- Autism spectrum disorders
- Schizophrenia
- Suicide and suicidal ideation

19. Public Health – Physical Health

- Physical health (general)
- Obesity
- Vomiting and nausea
- Chronic pain
- Spasticity
- Respiratory health
- Inflammatory diseases
- Sleep disorders
- Physical injuries and trauma
- Sexually transmitted diseases

20. Public Health – Child and Maternal Health

- Maternal health indicators
- Preterm births
- Infant mortality
- Other infant health markers

21. Public Safety – Crime and Justice

- Crime (general)
- Violent crime
- Property crime
- Public order crime
- Drug crime (general)
- Cannabis-specific crime
- Child maltreatment
- Incarceration
- Recidivism

22. Public Safety – Roadway Safety

- Cannabis-related crashes and fatalities
- Alcohol-related crashes and fatalities
- Drug-related crashes and fatalities (general)
- Traffic crashes and fatalities (nonspecific)
- DUI cannabis
- DUI alcohol
- DUI (nonspecific)
- Driving while texting and distracted driving
- Seatbelt and other safety feature use

23. Public Safety – Workplace Safety

- Workplace fatalities
- Workplace injuries
- Workplace safety violations and hazards

24. Socioeconomic – Business and Economy

- Economic productivity
- Corporate and industry valuation
- Research and innovation
- Business and regulatory fees
- Tax revenues
- Gambling revenues
- Travel and tourism
- Household expenditures
- Housing values and supply
- Social and economic equity

25. Socioeconomic – Labor Market

- Labor force participation
- Wages
- Work absences and sick days

26. Socioeconomic – Healthcare and Insurance

- Healthcare costs
- Disability claims
- Traffic accident insurance claims

27. Socioeconomic – Educational Attainment

- Student time use
- School attendance
- GPA/grades
- Degree completion

28. Socioeconomic – Environment

- Water theft and diversion
- Pollution
- Deforestation
- Wildlife endangerment

29. Socioeconomic – Demographics

- Interstate migration
- Birth rate and family size
- Minority population

30. Outcome Data Source

- Behavioral Risk Factor Surveillance System (BRFSS)
- Fatality Analysis Reporting System (FARS)
- Healthcare Cost and Utilization Project (HCUP)
- Monitoring the Future (MTF)
- National College Health Assessment II (NCHA-II)
- National Incident-Based Reporting System (NIBRS)
- National Longitudinal Survey of Youth (NLSY)
- National Poison Data System (NPDS)
- National Survey on Drug Use and Health (NSDUH)
- National Vital Statistics System (NVSS)
- Treatment Episode Data Set (TEDS)
- Uniform Crime Reports (UCR)
- Youth Risk Behavioral Study (YRBS)
- Other (specify)

31. Reference Population

- General population
- Criminal justice involved
- Crime victims
- Law enforcement
- Elementary school students
- Middle school students
- High school students
- College students
- Vehicle drivers
- Crash-involved individuals
- Decedents
- Cannabis users
- Tobacco users
- Alcohol users
- Prescription drug users
- Drug treatment clients
- Hospital patients
- Outpatients
- Poison center patients
- Medically insured
- Auto insured
- Disability claimants
- Medical professionals
- People with health conditions
- Workforce participants
- Property buyers/renters
- Urban population
- Internet/social media users
- Proprietary survey panelists
- Females
- Pregnant/postpartum women
- Newborns
- Children in need of protective services
- Parents
- Festival/nightlife participants
- Tourists and travelers
- Other groups (specify)

32. Reference Population Age-Group

- Children (0-5)
- Youth (6-11)
- Adolescents (12-17)
- Young adults (18-25)
- Adults (26-64)
- Older adults (65+)
- Not reported

**Methods**

33. Research Design

- Systematic review
- Cohort study
- Controlled before-and-after
- Differences-in-differences/fixed effects
- Interrupted time series
- Regression discontinuity design
- Synthetic control method
- Propensity score analysis/matching
- Instrumental variables estimation
- Cross-sectional
- Other design (specify)

34. Study Quality

- Minimal
- Low
- Moderate
- High
